# Cat brains age like humans: translating time shows pet cats live to be natural models for human aging

**DOI:** 10.1242/bio.062604

**Published:** 2026-06-22

**Authors:** Capucine Januel, Elijah Morrow, Ryan Gibson, Amanda Gross, Alexandra A. de Sousa, Brier A. Rigby Dames, Christine J. Charvet

**Affiliations:** ^1^ Department of Anatomy, Physiology & Pharmacology, College of Veterinary Medicine, Auburn University, Auburn, AL 36849, USA; ^2^ Ecole Nationale Vétérinaire de Toulouse, Toulouse 31076, France; ^3^ Scott-Ritchey-Research Center, College of Veterinary Medicine, Auburn University, Auburn, AL 36849, USA; ^4^ Centre for Accountable, Responsible, and Transparent AI (ART-AI), Department of Computer Science, University of Bath, Bath BA2 7AY, UK

**Keywords:** Translational, Aging, Development, Pet, Cat, Rate

## Abstract

Whether an animal can achieve a lifespan equivalent to a human in their 80s remains an open question. Cats may serve as valuable models for human aging because there is some evidence that they can develop human related aging patterns. Here, we leveraged 3754 observations extracted from age-related brain variation, blood chemistry profiles, and other data to equate ages across the lifespan of humans and cats. We used structural MR scans (7T and 3T MRI) from pet and colony cats to quantify age-related brain metrics during aging. Cat and human brains exhibit similar age-related patterns of brain atrophy. We used common patterns of brain change and other health-related metrics to generate age alignments across the lifespan to late stages of life (e.g. an 80-year-old human equates to a 15-year-old cat). We also collected observations across multiple cat populations, including pets, zoos, and colonies to encapsulate individual variation in cross-species age alignments. One major finding to emerge is that pet cats are studied at significantly older ages than colony cats, and pet cats demonstrate a high degree of age-related brain atrophy. We demonstrate that it is feasible to translate ages across the lifespan of humans and cats.

## Teaser

**Summary:** We analyzed 3754 observations to align developmental and aging trajectories across human and cat lifespans. We found similar patterns of aging across these two species, which suggests cats may be spontaneous models for aging.

## INTRODUCTION

**Fig. 1.**
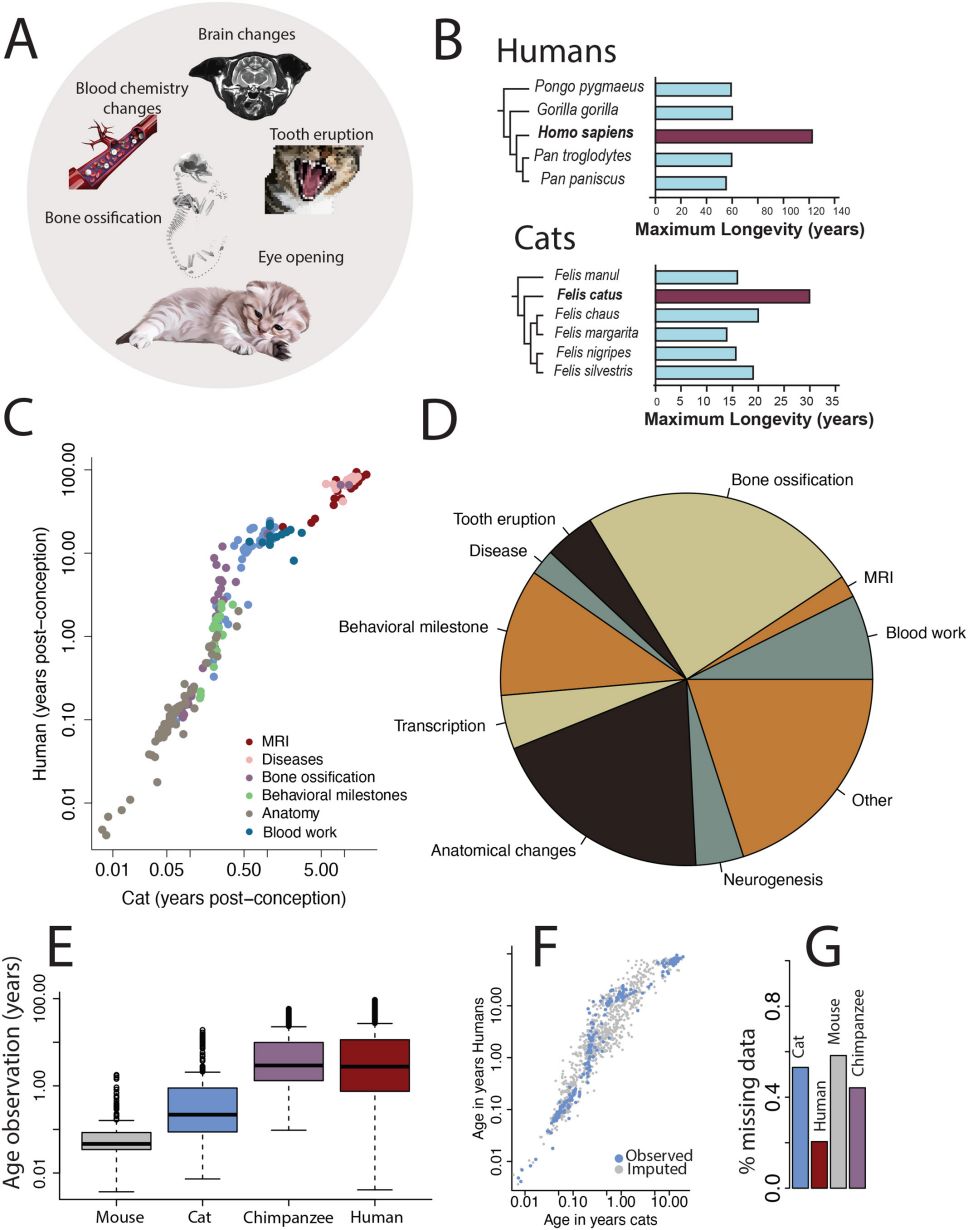
**(A) We used a range of metrics to align ages.** Those include age-related changes in brain structure, blood work, bone ossification, and behavioral milestones (e.g. eye opening). (B) Cats, like humans, have a lengthened maximum lifespan compared with closely related species. (C) Time points are color-coded according to data type. (D) Breakdowns of time points show their sources. A large fraction capture bone ossification, anatomical changes and a small percentage are from new MRI measures (1.9% of timepoints) and blood work (7.35% of time points). (E) Age range of unimputed observations cover much of the lifespan across different animals. (F) We imputed observations if they were not available to generate the model that finds corresponding ages across multiple species. An example is shown for cats and humans. (G) Percentage of missing data are shown for each species.

Human aging is characterized by cognitive and memory decline, brain atrophy, and increased incidence of geriatric diseases (Nelson et al., 2009; Long et al., 2012; Fjell et al., 2013; Coupé et al., 2019). It was thought that such age-related changes were unique to humans (Finch and Austad, 2015), though we are increasingly finding that animals develop brain amyloid pathology and tangles (e.g. cats, chimpanzees; Youssef et al., 2016). It is still an open question as to whether other animals share patterns of brain atrophy that resemble humans and whether they live sufficiently long to recapitulate age-related diseases (Yokoyama et al., 2022; Rigby Dames et al., 2023; de Sousa et al., 2025; Kraska et al., 2011; Picq et al., 2012; Chen et al., 2013, 2013; Rosen et al., 2008; Edler et al., 2017; Charvet, 2021; Charvet et al., 2023). Here, we collected a wide range of metrics (Fig. 1) to generate cross-species age alignments across the lifespan of humans and cats and tested whether cats share similar patterns of brain aging with humans.

Cats can live relatively long lives and there is some evidence they may share aging patterns with humans (Freeman et al., 2006; Sordo et al., 2020, 2021; Rofina et al., 2006; Landsberg et al., 2012; Chambers et al., 2015; Seibert, 2017; Sordo et al., 2021). According to AnAge, the maximum lifespan in humans (i.e. 122.5 years) is nearly twice that of great apes (e.g. 68 years in chimpanzees; de Magalhães and Costa, 2009; de Magalhães et al., 2024; Fig. 1B). Domestic cats, like humans, also have a longer maximum lifespan (*Felis catus*, 30 years) than closely related species (e.g. wildcat; *Felis silvestris*, 19 years; Tacutu et al., 2013; Teng et al., 2024; Fig. 1B). Also, there are an estimated 600 million cats worldwide (Driscoll et al. 2009a,b; Griffin and Baker, 2007). We focus on cross-species age alignments across the lifespan of humans and cats with a particular focus on aging because cats live long lives and they are numerous, suggesting we may study them in large enough samples to assess whether their ages can be mapped onto a human in their 80s and beyond.

We extracted corresponding time points from common patterns in behavior and biological change, including abrupt transformations, age-related changes in blood work and brain structure (Fig. 1; Charvet et al., 2025; Cottam et al., 2025). These observations were selected to span a broad age range so that we could equate ages across the lifespan. This work builds on a project called Translating Time (www.translatingtime.org), which equates ages across species. We used cats from different environments (e.g. pets, colony, zoo) to generate cross-species age alignments that are relevant to cats from different environments. We found that pet cats are studied at significantly older ages than those housed in colonies, which suggests that the study of pet cats can inform human aging.

## RESULTS

**Fig. 2.**
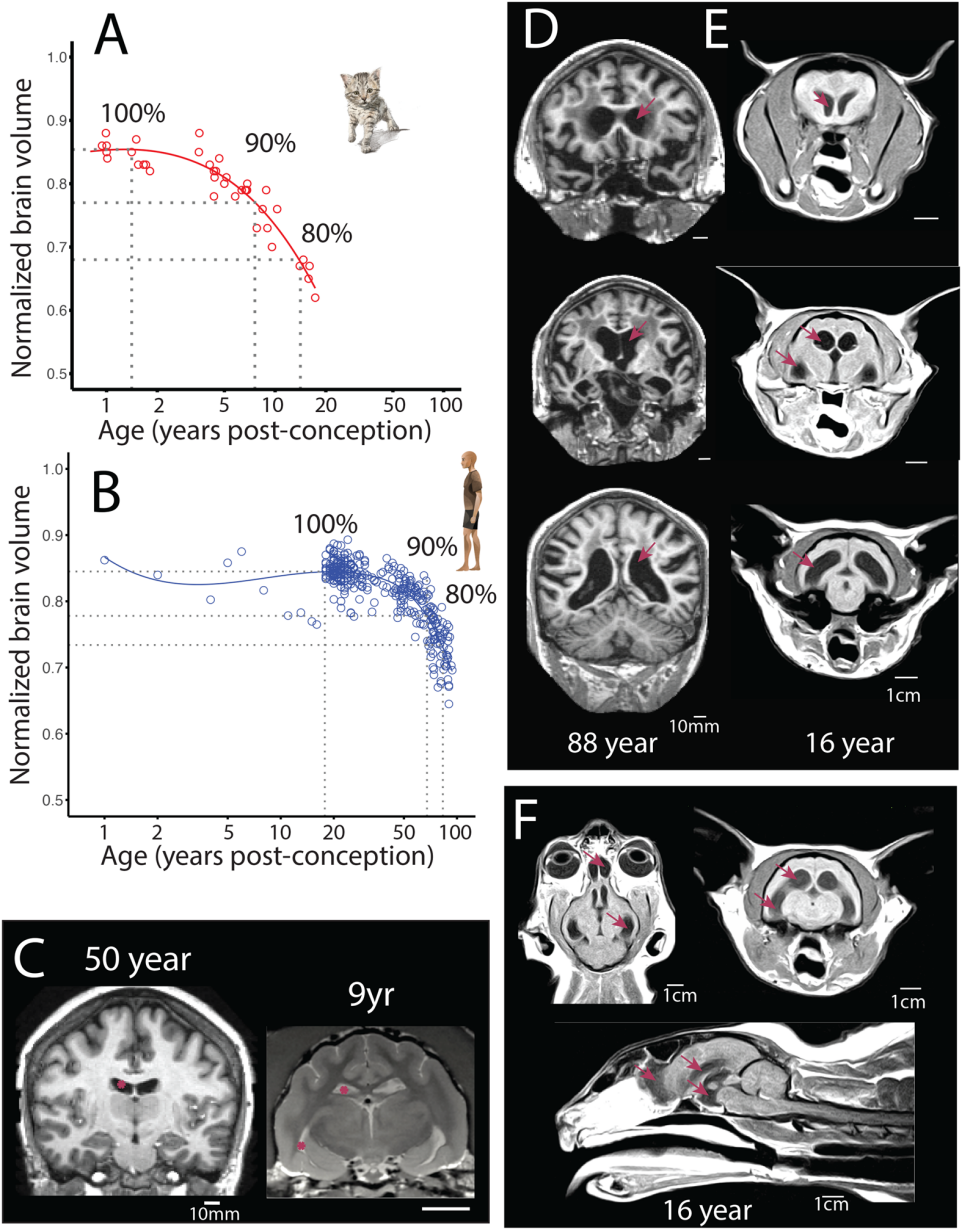
**The brain atrophies with age in (A) cats and (B) humans.** We fit a smooth spline to multiple metrics (e.g. normalized brain volume) versus age to extract corresponding time points. We, for example, extracted the age of peak brain volume (100%) before the brain begins to decline and the age at which the volume reaches a particular percentage of adult brain volume (e.g. 90%, 80% of maximum brain volume). We use these time points to generate cross-species age translations. (C) A 50-year-old human (C) and a 9-year-old cat (C) begin to show modest enlargement of ventricles (red asterisks). At later ages, the ventricles are much enlarged (arrows) as exemplified in an 88-year-old human (D) and a 16-year-old cat (E). (F) Horizontal, coronal and sagittal slices show the extent of the brain atrophy in the same 16-year-old cat, which show expanded lateral and third ventricles across the olfactory bulb and telencephalon (arrows).

We used 3754 observations from multiple sources, including age-related changes in brain structure, blood chemistry profiles, disease onset and progression, as well as abrupt transformations from cats, humans, and other species (Fig. 1A-D; Fig. 2; Tables S1-S6; Charvet, 2021; Charvet et al., 2023; Cottam et al., 2025). We use these data to generate a model to equate ages across species (Datasets 1-6). We first discuss how we use harnessed time points from age-related variation in brain structure in humans and cats. We subsequently discuss how we collected observations from blood chemistry profiles and how we integrated these observations to generate cross-species age alignments across the lifespan (Figs S1, S2).

### Cat and human brains atrophy with age

**Fig. 3.**
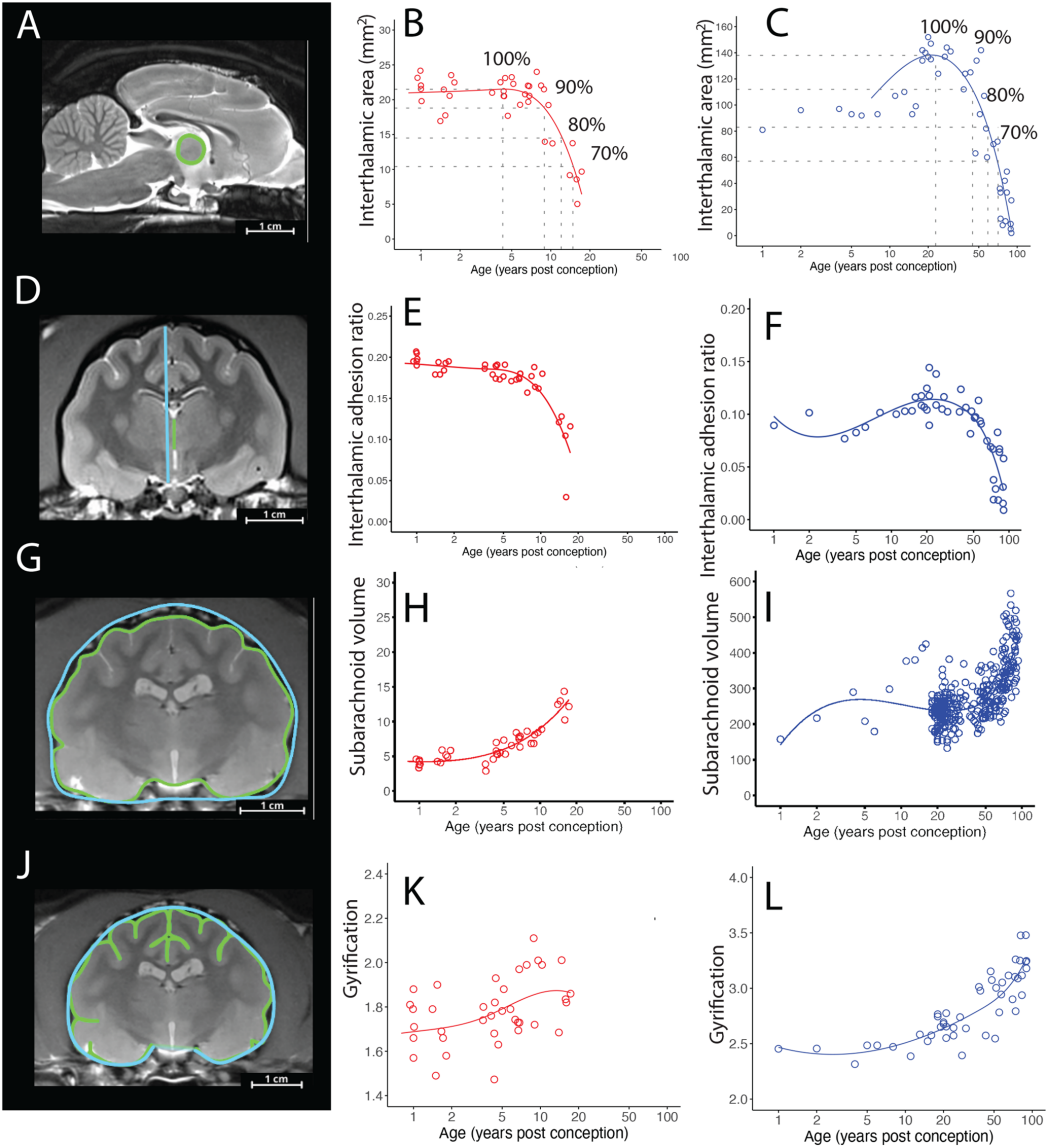
**Sagittal and coronal slices from 7T MRI brain scans illustrate some of the brain measurements.** (A) We used sagittal slices to measure the interthalamic area (color-contoured). The interthalamic adhesion area peaks and declines with age in (B) cats and (C) humans. We fit smooth splines to capture ages of peak interthalamic adhesion area before it declines to specific percentages (e.g. 90%, 80%, 70%) of its maximum size (B,C). We used coronal slices to measure the interthalamic adhesion relative to the brain height (D-F). The interthalamic adhesion ratio declines with age in (E) cats and (F) humans. (G) We also measured the cranial space, which is the volume of the brain subtracted from the cranial box. To do this, we measured the brain and cranial box (color-contoured; G) from coronal slices. The intracranial space called subarachnoid volume increases with age in (H) cats and (I) humans. (J) We quantified gyrification as the ratio between the perimeter contouring gyri and sulci relative to the brain's contour. Gyrification increases with age in (K) cats and (L) humans. The cats are 4.45 years (A) and 8.85 years post-birth (D,G,J).

Brains become atrophied with age in cats (Fig. 2A) and humans (Fig. 2B) and this atrophy is concomitant with relatively enlarged ventricles (e.g. 16-year-old pet cat; Fig. 2D-F). The feline pattern of brain atrophy mirrors those of humans in their 80s (Fig. 2D-F). We fit smooth splines to normalized brain volume versus age and brain structures (e.g. interthalamic area, subarachnoid volume, gyrification) to compare age-related trends in humans and cats (Fig. 3; summary statistics in Table S5). Age-related changes follow similar trends in humans and in cats whether structures increase with age (e.g. subarachnoid volume) or if they decrease with age (e.g. interthalamic adhesion). We used smooth splines through the brain, as well as the interthalamic adhesion area and thickness to extract corresponding time points in humans and (Fig. 3). We extracted the age at which the brain volume is maximal (i.e. 100%) and when it reaches a percentage of its maximum size (e.g. 90%, 80%) in the two species. We repeated this approach for the interthalamic adhesion area and thickness, which showed similar age-related changes in humans and cats.

### Pet cats receiving MRIs are older than colony cats

**Fig. 4.**
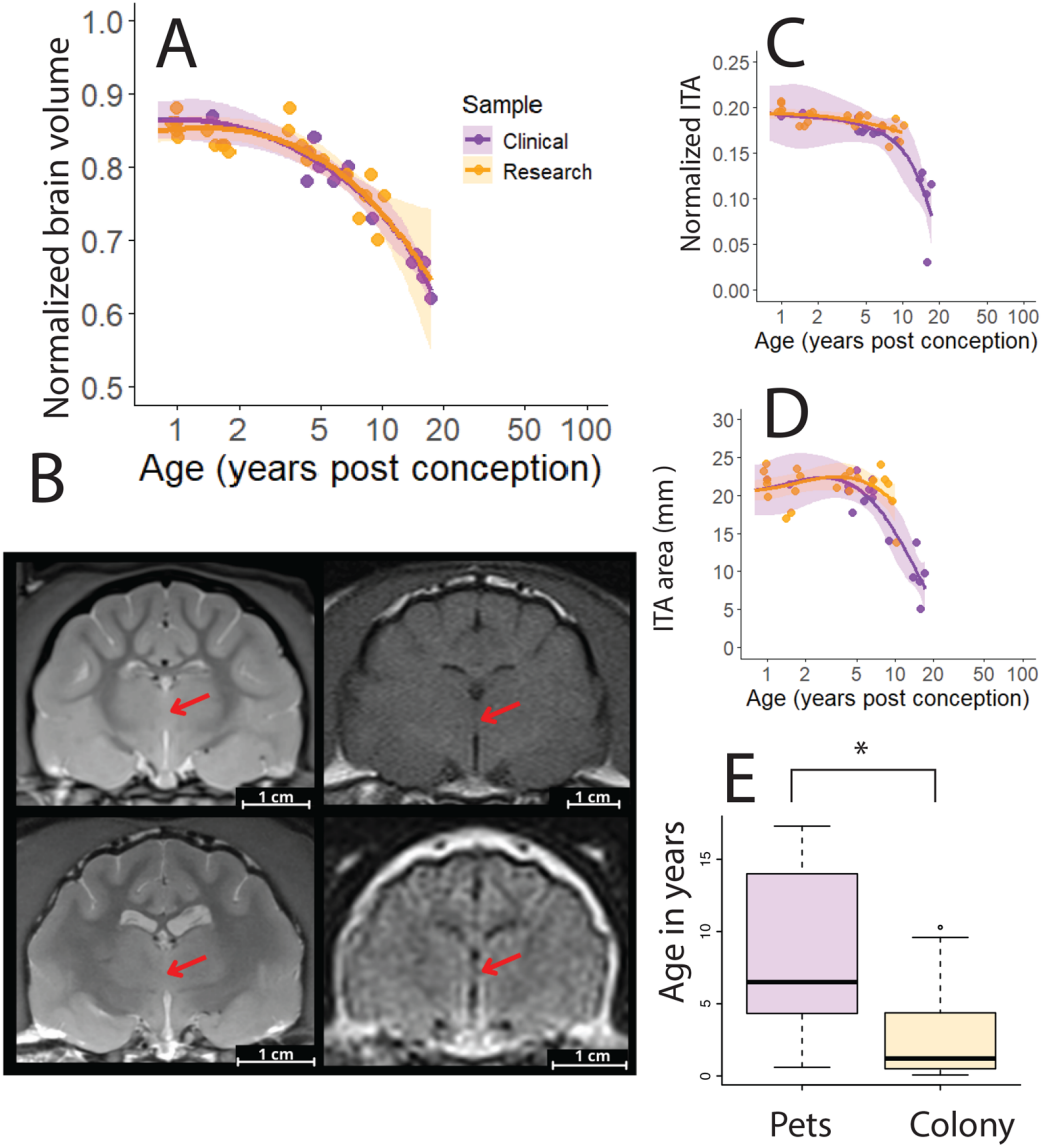
**(A) Normalized brain volume declines with age in both colony and pet cats.** (B) Coronal slices show example images collected with the 7T MRI scanners (left panels) and the 3T MRI scanners (right panels) in B. (A,C,D) We fit smooth splines and their confidence intervals to Auburn colony and pet cats separately, and we found extensive overlap in brain metrics across these two feline populations. Colony cats were scanned in a 7T MRI, which can generate scans of higher resolution than pets visiting the clinic. Brain metrics, including the normalized interthalamic adhesion thickness (C) and the interthalamic adhesion area (D) are also very similar across colony and pet cats. (E) Pet cats visiting the clinic are significantly older than those in laboratories. We used the highest resolution MR imaging modality, which means we used T1 or T2-weighted images. y, years after birth. (B) These cats varied in age (top left; 0.62 year; bottom left: 8.85 years; top right: 4.66 years; bottom right: 17 years). ITA, interthalamic adhesion area. *, statistically significant.

We compared pet and colony cats to evaluate whether population or MRI contributes to variation in brain metrics (Fig. 4A-D; Fig. S1). We fit smooth spline models to brain metrics versus age separately for pet and colony cats (Fig. 4). The smooth splines are within each other's 95% confidence intervals, which means that patterns of brain atrophy in pet and colony cats overlap substantially. We did find one important difference between clinic and colony. Pet cats visiting the clinic for MRIs (x=7.81 years old; *n*=34; Kolmogorov–Smirnov test: D test=0.569, *P*≤0.01, *n*=18, *n*=34; Fig. 4E) were significantly older than those in colonies (x=2.77 years old; *n*=18). The age of colony cats generally extended up to approximately 10 years, though cats visiting the clinic were in their teens. Accordingly, pet cats show the most atrophy in their brains (Fig. 4A).

### Diverse feline populations are used to translate ages across species

**Fig. 5.**
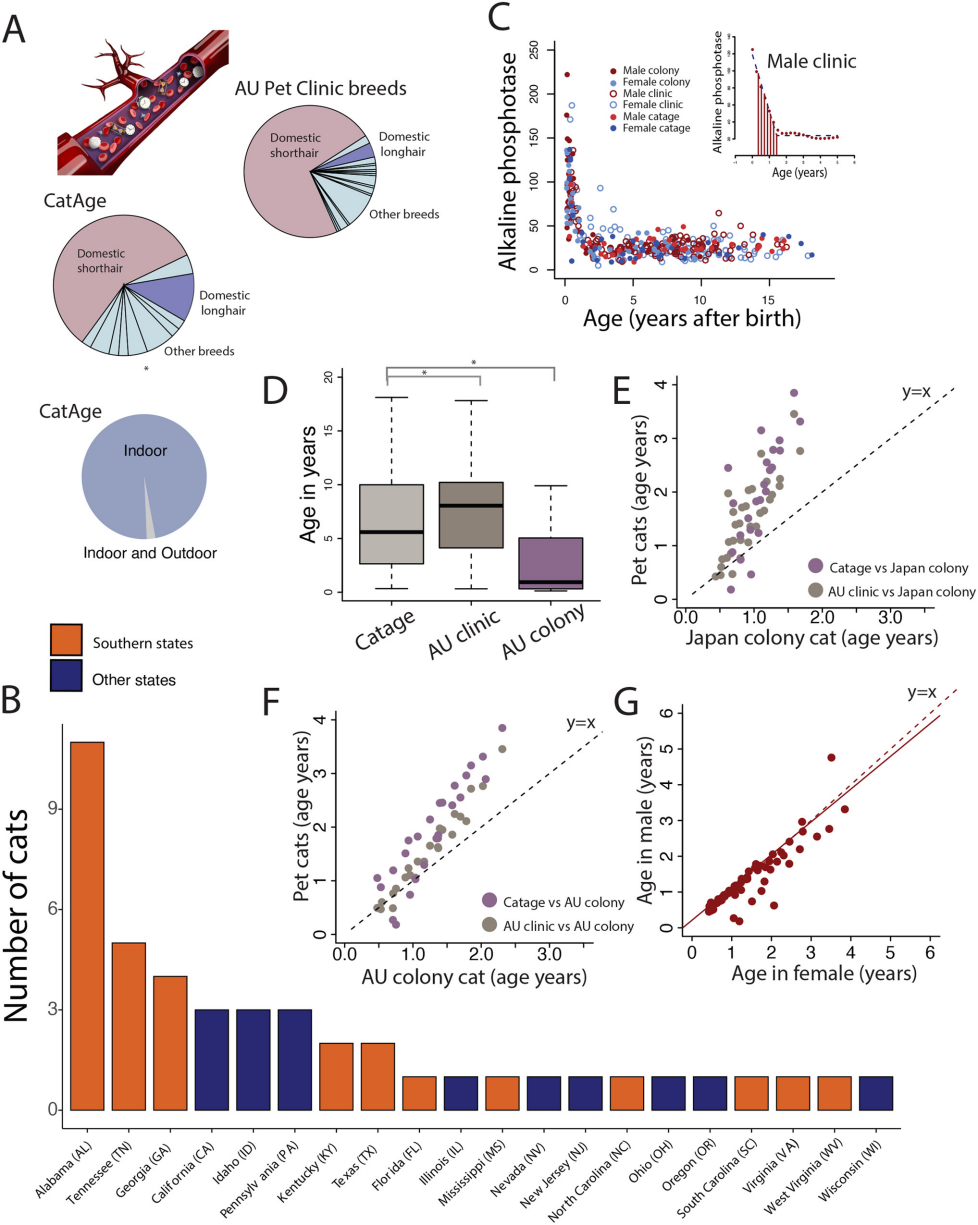
**We used blood chemistry profiles to generate age alignments across species.** (A) Most of the cats used in this study are domestic shorthairs. (B) Cats from Project CatAge are primarily from southern states, but the dataset includes representations across the USA. (C) ALP varies with age and shows common patterns of change in cats from the colony, the Auburn University clinic, and Project CatAge. We first fit a smooth spline through these data followed by a non-linear model (see inset) to extract the age of plateau as well as epochs (e.g. 90% of plateau) within multiple cat groups, including colony cats housed at the Toxicology laboratory in Kyoto, Japan (Nakai et al., 1992) and one housed at Auburn University (Nakai et al., 1992). (D) Cats from the Auburn University clinic (*n*=169) and Project CatAge (*n*=45) are significantly older than the colony cats (*n*=99). Here, we applied a non-parametric Welch one way test followed by a Games-Howell test to the age of cats for which ALP was measured. An asterisk denotes the pair-wise tests were significant (*P*<0.01). (E-G) Comparative analysis in time points extracted from blood work values show that pet cats take longer to mature than colony cats. (E) This is evident when comparing all pets to all colony cats though there is no effect for (G) sex. Pairwise comparisons between pets versus the (E) cats housed in the Japanese colony and the (F) Auburn University colony consistently show that pet cats take longer to mature than colony cats. Data are presented as box plots where the center line denotes the median, the box represents the interquartile range (25th to 75th percentiles), and the whiskers represent the minimum and maximum values.

**Fig. 6.**
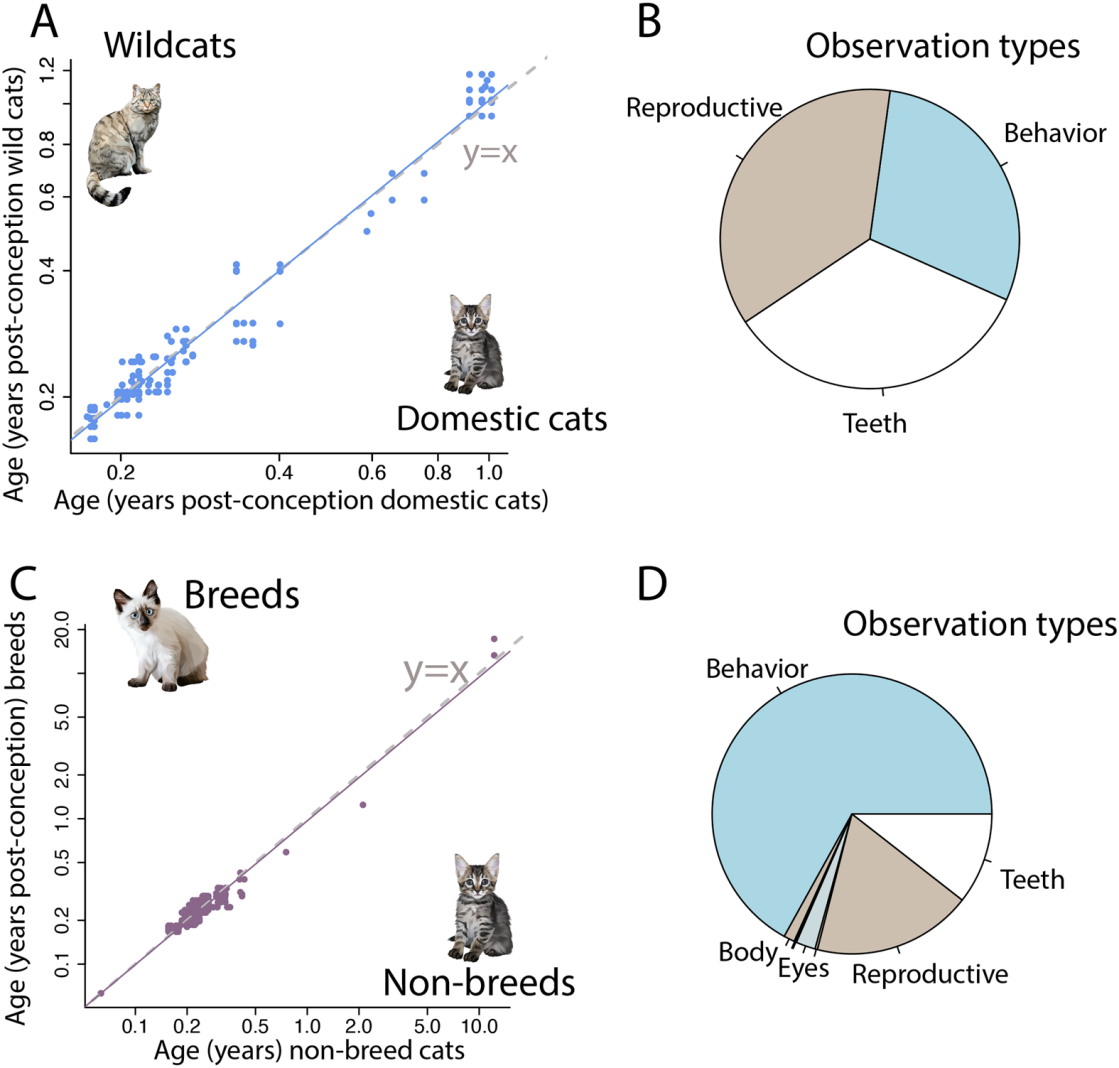
**(A) Domestic cats and wildcats mature at similar rates.** The fit to the model has a slope close to 1 (y=x). Observations (e.g. age of eye opening) were matched for sex, environment, and statistics (e.g. minimum age of eye opening). (B) These observations capture the pace of behavioral, reproductive, and tooth development. (C) Breeding is not associated with modifications in the pace of development. A linear model fit to breed, and non-breed cats shows that the linear model is very close to 1. (D) Observations are primarily from behavioral, reproductive and tooth maturation.

**Fig. 7.**
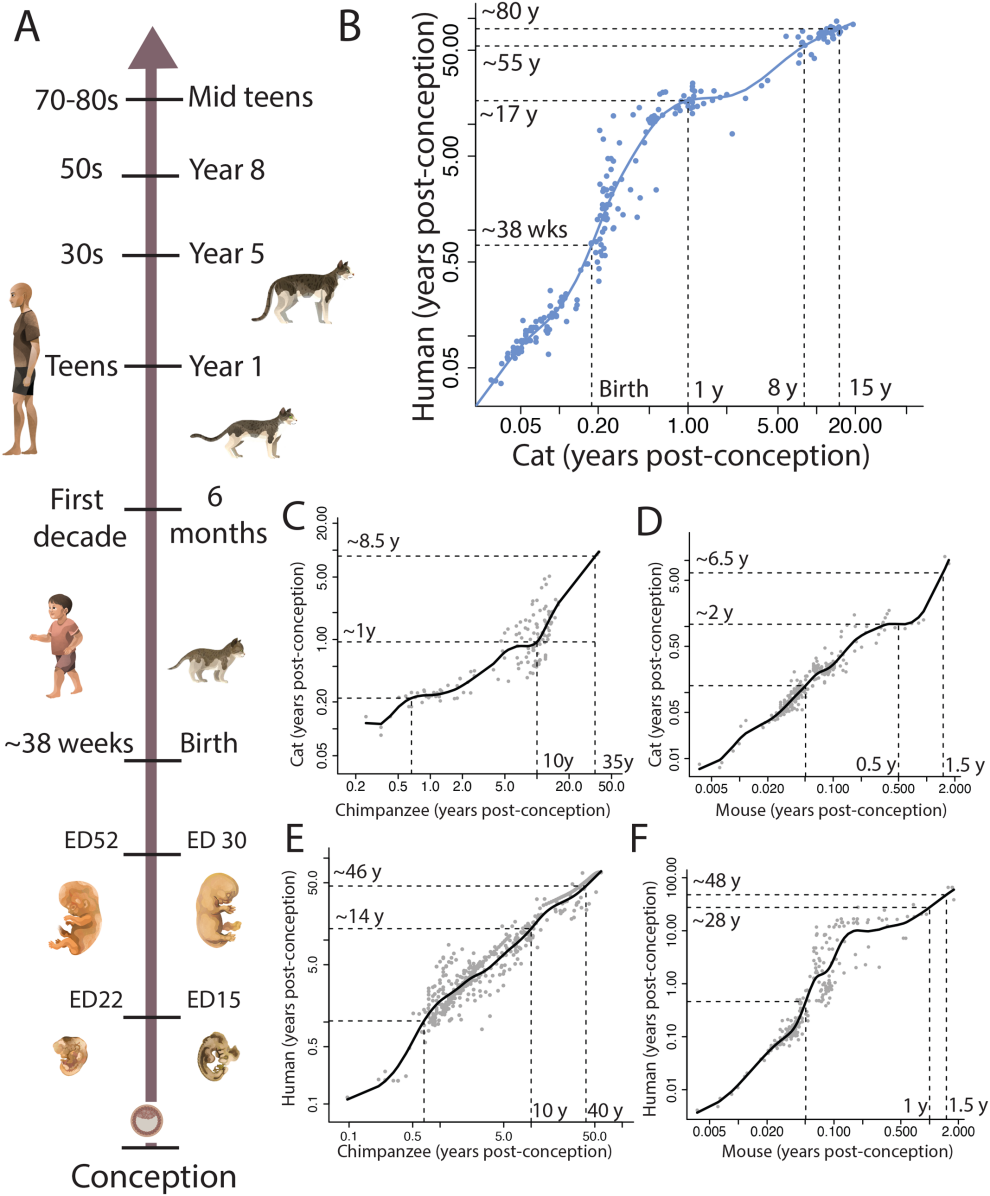
**Example age matches across species.** (A) A newborn cat equates to a human at 40 weeks post-conception, and a 6-month-old cat equates to a human within their first decade, and a cat in their teens maps onto a human in their 80s. (B-F) Scatterplots of two species comparisons demonstrate that there is a complex relationship in the pace of development and aging across species. Dashed lines show examples of corresponding ages between two species.

**Fig. 8.**
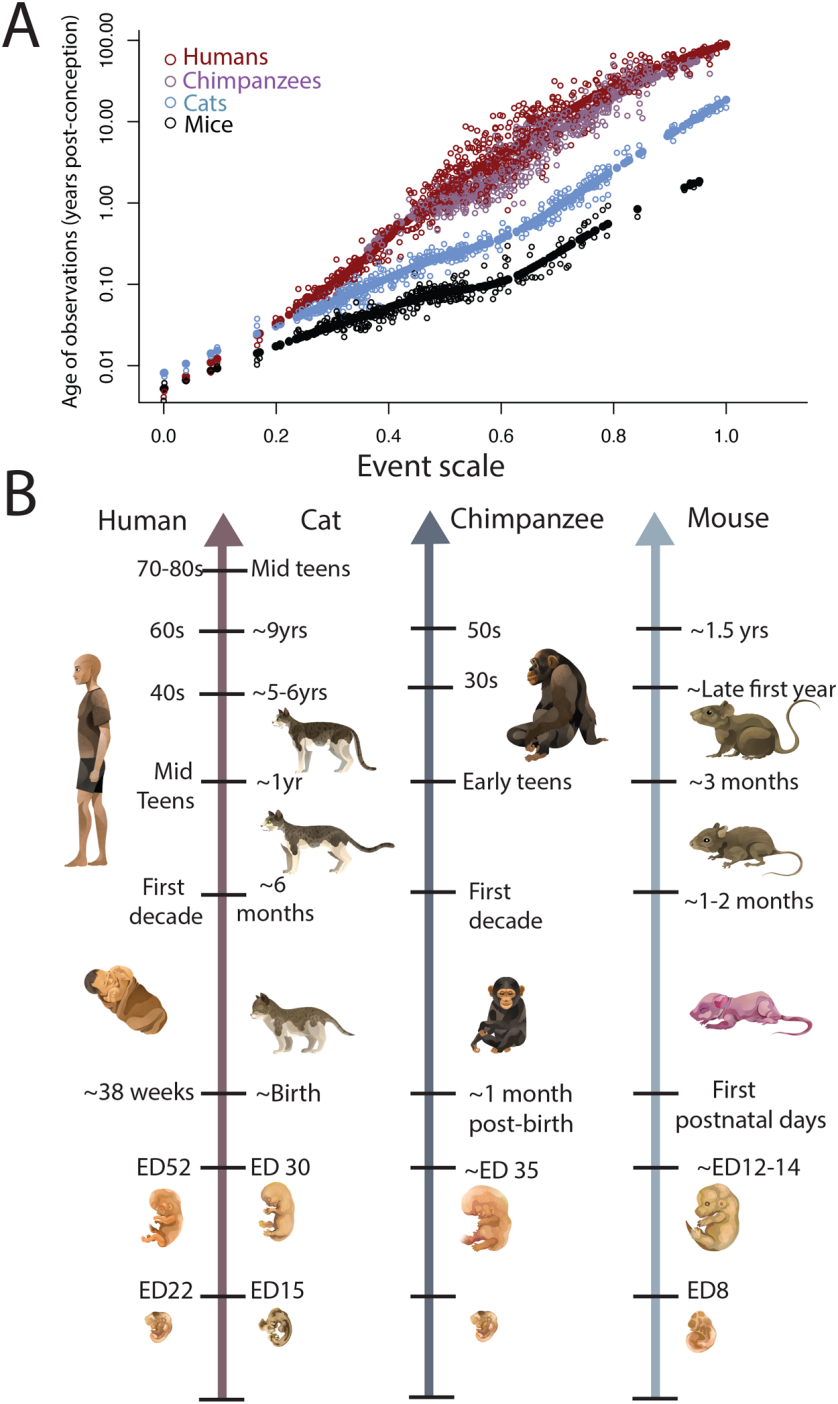
**We used a linear model to translate ages across species.** (A) Time points expressed in years post-conception are plotted against the event scale. Open circles represent observations whereas closed circles represent predicted values. The smooth spline allows for variation in the pace of development and aging. (B) Here are some examples of age alignments generated from the model. PM, postnatal month; P, postnatal day.

We used age-related variation in health metrics (e.g. body weight, blood chemistry profiles) and behavioral milestones (e.g. eye opening) from multiple sources (Table S1). We used these data to generate cross-species age alignments and to compare the pace of development across species (Figs 6-8; Table S1). We focused on health metrics because we could obtain information on cats from multiple sources (Auburn colony, the Auburn clinic, Project CatAge) and because these metrics vary with age. For example, alkaline phosphatase (ALP) declines postnatally to relatively stable levels (Fig. 5). We fit smooth splines followed by non-linear regressions to health or somatic metrics (i.e. ALP, total protein, phosphorus, body weights) to detect the age of plateau and the ages at which specific percentages of that plateau value are reached (Fig. 5C-F). We observed variation in rates of development across cats. For example, pet cat somatic maturation (linear model: F=339.5, R²=0.76, *P*<0.01) proceeds more slowly than those of colony cats. The slope of the linear model (y=1.77x−0.23, adj R^2^=0.76; d.f.=104; *P*<0.01) fit to these data are significantly greater than 1 (slope=1.77; t=7.99; *P*<0.01; Fig. 5E,F). Our model incorporates the variation in the pace of development of cats from various environments by averaging these data before generating cross-species age alignments.

### Pet cats visiting clinics for their health were significantly older than colony cats

Pet cats (from the Auburn clinic and CatAge) receiving blood work analyses were significantly older than colony cats (Fig. 5D; non-parametric Welch's test on ALP: F=76.235, *P*<0.01; Games–Howell post hoc test; *P*<0.01; Fig. 5D). Colony cats were studied up to roughly 10 years of age whereas pet cats underwent health assessments in their late teens (Fig. 5D). Therefore, pet cats represent an older population than colony cats.

### Domestic cats and wildcats are similar in their pace of development

We amassed observations across diverse cat groups, including wildcats (*F. silvestris*) and domestic cats (*F. catus*) to test whether they differ in their pace of development (Fig. 6A). Observations were matched for statistics (e.g. minimum age of eye opening), sex, and environment (e.g. colony captive, captive, wild; Table S1). The pace of development in domestic cats (excluding breeds) was not significantly different from wildcats (Fig. 6A). The slope (1.02; s.e.=0.014; t=1.43; d.f.=154, *P*=0.155; Fig. 6A) of the linear model fit to the log-transformed age (y=1.02+0.008; adj R^2^=0.97; *P*<0.01, d.f.=154; predictor: domestic cat) was not significantly different from 1. The pace of development in cat breeds is like non-breed cats. The slope of the linear model fit to log-transformed years post-conception for non-breed cats versus breeds (y=0.99x−0.01; adjusted R²=0.93; d.f.=709; Fig. 6B; predictor variable: non-breed cat) was not significantly different from 1 (slope=0.98±0.01 s.e.; t=−1.07; *P*=0.29). Our findings suggest that there was little change in the overall pace of development during domestication (Datasets 3-6).

### Translating time across the lifespan of humans and cats

We used health indicators, age-related variation in brain structure, and other metrics to generate cross-species age alignments (Figs 7,8). We integrated data from humans, cats, chimpanzees as well as mice because we previously gathered time points across their lifespan (Charvet et al., 2023; Cottam et al., 2025). The inclusion of these species bolsters our insights into the evolution of development and aging rates. We imputed missing data, and we generated an event scale (Fig. 8), which is an ordering of time points. For the event scale, early time points were assigned a score close to 0 and later time points were assigned a score close to 1 (Fig. 8A; Dataset 1). We used a smooth spline basis expansion to enable a flexible fit of the data to the event scale (Fig. 8A) because we observed relative accelerations in the pace of development in some species.

### Translating time shows the pace of development and aging follows a complex trajectory

We fitted a general linear model with the event scale as the independent variable and observations as the dependent variable. The model with a spline expansion accounts for a significantly high percentage of the variance (F=3905, *P*<0.01, adj R^2^=0.977; d.f.=2077; Fig. 8A). The model (Fig. 8) along with scatterplots of unimputed observations (Fig. 7) showed that postnatal development is relatively prolonged in humans and chimpanzees compared with cats and mice (Fig. 8A). Humans and chimpanzees take relatively longer to reach adulthood than cats and mice. Scatterplots of unimputed observations further highlight this variation (Fig. 7). Humans in their 80s map onto cats in their mid-teens (Fig. 7A), but not every species maps onto a human octogenarian (Fig. 7C-F). For example, chimpanzees in their 40s map onto humans in their mid-late 40s (Fig. 7C). Because few chimpanzees live past their 40s, it is difficult to generate age alignments in chimpanzees past this age. As another example, a 1.5-year-old mouse maps to a human at 48 years of age, though few mice live past their second year (Fig. 7F). In contrast, cats live into their teens, which corresponds to humans in their 80s (Fig. 7A,B).

## DISCUSSION

We collected observations from multiple metrics, including age-related variation in brain structure and health metrics to translate ages across species. We found common patterns of brain atrophy across humans and cats. We used diverse feline populations to encapsulate individual variation in cross-species alignments. We found that pet cats are studied at older ages than colony cats, making their data most relevant for human aging research. We discuss how studying pet cats can improve our understanding of human aging.

### The pace of development and aging is complex

Our work shows that multiplying a species' age by a factor is not a satisfactory means to equate ages across species. Rather, certain phases of the lifespan are relatively stretched or compressed depending on life stage or studied species. For example, humans possess a relatively extended postnatal period of development compared with cats and mice (Figs 7,8). This is in contrast with what is observed in chimpanzees where the human pace of postnatal development is like chimpanzees with no obvious extension in the duration of human postnatal development (Figs 7,8; Charvet, 2021; Charvet et al., 2023). The most parsimonious interpretation of these data is that a lengthening of postnatal development evolved with or before the emergence of hominids. Several animals we study to inform health such as mice, rats or cats have a relatively accelerated rate of postnatal development (Cottam et al., 2025).

### Cats live to the equivalent of a human octogenarian

We relied on multiple metrics to translate ages across species (Fig. 1). This multi-metric approach enabled us to translate ages across the lifespan of humans and cats with humans in their 80s mapping onto cats in their teens. This is important because not every animal lives to the equivalent of a human octogenarian. For example, few chimpanzees live past their 40s, which equates to humans in their 50s (Charvet, 2021; Charvet et al., 2023). Sample size is a major challenge in the field of aging. In the laboratory, long-term care is demanding, and few individuals survive to advanced ages. While these challenges are characteristic of laboratory research, some pet cats reach old age, and they visit veterinary clinics and can be studied with a range of tools (Fig. S1A, blood work, MRI). Therefore, pet cats are an accessible aged population that could inform human aging research (Sánchez-Vizcaíno et al., 2017; Mata, 2025).

### Cats and humans share age-related changes in brain structure

We found that humans and cats share similar age-related changes in brain structure. These observations add to the list of age-related traits cats and humans share, which include cataracts, degenerative joint diseases, as well as cerebral cortex plaques and tangles (i.e. 14.1 years post-birth; de Sousa et al., 2025; Gunn-Moore et al., 2007; Gunn-Moore, 2011; Sordo et al., 2021). Pet cats showed more brain atrophy, and they were older than colony cats. Because colony cats were not studied past their teens, we were not able to compare age-related brain atrophy between cat groups. We were not able to assess whether MRI, environment, or health status impacts rates of brain atrophy. Future work will be needed to identify whether environment, cognitive dysfunction, or genetic background may influence rates of brain atrophy across feline populations. We have much more to learn about feline cognitive dysfunction. Surveys and assessment tools exist to evaluate cognitive dysfunction in cats, but we have yet to develop cognitive assays that specifically test for memory impairment (Landsberg et al., 2010; Gunn-Moore, 2011; MacQuiddy et al., 2022). The development of such tests are needed before we can evaluate whether brain aging is accelerated in cats with cognitive dysfunction.

### Aged pet cats inform human aging

We found that pet cats were studied at significantly older ages than those housed in colonies. While these observations, by themselves, do not mean that the lifespan of pet cats is longer than cats housed in colonies, they do show that there is a natural propensity to study aged pet cats. Studying aged animals in the laboratory is challenging because animals need to be housed for a significant amount of time (e.g. 15 years) for them to age. In contrast, pet cats represent a viable group of aged animals available for study. Pet owners fund expensive diagnostics (e.g. MRIs) with the aim of diagnosing and treating diseases, and this interest could be leveraged to advance our understanding of human aging (Gunn-Moore, 2011; Chambers et al., 2015). Our study lays the foundation for future work to evaluate how cognitive dysfunction impacts feline aging.

### Improving animal health to understand human health

Much research has focused on laboratory animals even though findings from laboratory animals often fail to translate to humans. This is presumably because diseases are artificially created in the lab, they are studied in environments and ages that are not relevant to humans (de Sousa et al., 2025). Our past work translating ages between humans and mice suggests that very few mice live to the equivalent of a human octogenarian (Cottam et al., 2025). Pet cats may inform human aging since humans and cats age similarly and they develop health challenges that mirror those observed in humans.

## MATERIALS AND METHODS

We integrated observations with previously published data to equate ages across species (Figs 1,2; Clancy et al., 2001; Workman et al., 2013; Charvet and Finlay, 2018; Charvet et al., 2022; Cottam et al., 2025; Table S1). Whereas an ‘observation’ focuses on a single datum point, a ‘time point’ refers to several observations recorded in at least two species (e.g. age of eye-opening). Some time points are specified by sex (e.g. reproduction, body growth), while other time points are drawn from both males and females (Table S1). The ‘Male+Female’ designation is used for data from both sexes as well as for cases where the sex was not recorded. Time points are associated with summary statistics. We considered the minimum (value=1), midrange, median, mean (value=2) or maximum age (value=3). If the statistics were unknown, we set it to 2. Individuals may be pets (i.e. captive clients), from colonies (i.e. captive laboratories), or from an unknown environment. Colony cats are defined as animals that are maintained in an enclosed area for research and breeding. Pet cats are companion animals that are studied in clinical, laboratory, shelter, or home settings. Zoo cats are managed for species preservation rather than for human companionship and are housed in enclosures. Missing data were imputed to generate the event scale used in the model to translate ages across species (Fig. 8; Table S2). The scatterplots showing two species comparisons are from unimputed observations (Fig. 7). We used Web Plot digitizer v. 4 to extract data from published plots, particularly for data points located between axis tick marks. All statistical analyses were performed with the programming language R (Datasets 1-4). Table S1 lists individual observations, specifies sex, statistics, and the environment. Table S2 lists averaged time points per species with missing data denoted by ‘NA’.

### Age distribution of pet cats from the Auburn clinic

The ages of cats in this study were constrained by availability. There was a sharp decline in the number of cats visiting the clinic after 12 years of age (Fig. S1A). The inclusion of pet cats visiting the clinic permits studying individuals in their teens but is also limited by the fact that relatively few individuals visit the clinic at advanced ages (Fig. S1A; Figs 7,8). We did not attempt to recruit aged cats for MRI studies because cats needed to be sedated to acquire MRI scans, which carries a heightened risk of mortality in aged animals.

### Cat brain MRI

We used multiple MRIs machines (3T, 7T MRI scanners) to study brain structure in cats housed in the colony and from pets visiting the Auburn clinic. These MRIs were collected for purposes other than the present study, which obviates the need for IACUC approvals. We used MRI scans from cats from the Auburn veterinary clinic (ages: 4.33 months-17.3 years of age post-birth; *n*=18 MRI scans; 12 males, six females), and colony cats (ages: 0.07-10.29 years after birth; *n*=34 MRI scans; ten males, 24 females; mean age: 2.53 years after birth). We repeated measures for some individuals. A board-certified veterinary neurologist evaluated health records and MRIs to exclude pet clinical cats with brain lesions. These morphometric analyses are based on 52 cats (22 males, 30 females) and 446 human participants (*n*=171 males; 275 females). We evaluated whether the MRI type impacts metrics (Fig. 4) and whether the inclusion of observations derived from MRI fall in line with other observations (Fig. S1B).

### Cognition

A board-certified veterinary neurologist evaluated the health records of pet cats receiving MRIs in the clinic and confirmed a lack of evidence of cognitive dysfunction syndrome in studied cats. We considered behavioral changes, such as spatial or temporal disorientation, vocalizations, changes in interactions with others and sleep-wake cycles, activity levels, house-soiling, anxiety, learning and memory deficits in these cats (Sordo et al., 2020). Researchers overseeing the cat colony did not observe signs of cognitive dysfunction. Therefore, we consider that age-related changes in brain structure reflect normal aging, though we cannot rule out the possibility that some cats may have cognitive dysfunction.

### 7T MRI scanner

We used the 7T MRI Siemens MAGNETOM clinical scanner. Cats were scanned as part of other long-term efforts to study lysosomal storage disease (Gray-Edwards et al., 2014). MRI scans were collected with a 28ch Rx×1ch Tx knee coil (QED). Cats were anesthetized with dexmedetomidine (0.04 mg/kg) and ketamine (10 mg/kg) intravenously. After brain scanning, general anesthesia was discontinued, and dexmedetomidine was reversed with antisedan**.** Multiple MRI pulse sequences were collected, which include MPRAGE (magnetization prepared rapid acquisition gradient echo), SPACE (sampling perfection with application optimized contrasts using different flip angle evolution), T2 TSE (turbo spin echo), HASTE (half-Fourier acquisition single-shot turbo spin echo) and CEST (chemical exchange saturation transfer). We used either T1 or T2 *in vivo* MR images. TR, TE (e.g. TR=50 ms, TE=4 ms; TR=5450 ms, TE=12 ms), and voxel resolution varied across scans. Some scans were anisotropic (e.g. 0.336 mm×0.336 mm×1.2 mm), and others were isotropic (e.g. 0.5 mm×0.5 mm×0.5 mm).

### 3T clinical MR scanner

Cats in the clinic were anesthetized and scanned with a 3T MRI Siemens Skyra MRI Scanner. The standard procedure is to administer pre-anesthetic medication followed by induction and gas anesthesia with isoflurane before scanning. Scan resolution (anisotropic resolution: e.g. 0.469 mm×0.469 mm×3.3 mm; 0.781 mm×0.781 mm×5 mm) and imaging parameters (e.g. TR=600, TE=11.5; TR=2000, TE=10) varied across scans. During these sessions, T1 and/or T2 scans were collected. We used the images with the highest resolution for our morphometric analyses. A board-certified neurologist selected MRI scans from the clinic and excluded cats with brain lesions or brain masses. We do not use templates because the MRI scan image quality and types varied across individuals, but we did compare brain scans across groups to assess whether the MRI impacted measurements (Fig. 4).

### Human brain scans

We used structural MRI scans of human brains (ages: 1-99 years old) from multiple databases, including OASIS1 (MPRAGE sequence; anisotropic resolution; 1 mm×1 mm×1.25 mm), Brain Chart, and NITRC (Bethlehem et al., 2022; Richards, 2019). Details of T1- and T2/FLAIR-weighted structural MR scans were described previously (Marcus et al., 2007; Bethlehem et al., 2022; Richards, 2019; Fotenos et al., 2005). We used the Clinical Dementia Rating (CDR) score (Berg et al., 1998), which is a measure of dementia across multiple cognitive domains (e.g. memory, orientation, judgment, personal care). We selected individuals with a CDR score of 0, which indicates a lack of evidence for dementia.

### Volumetric analyses

Normalized whole brain volume (nWBV) was computed by dividing brain volume (omitting the lateral ventricles) by the cranial box. To obtain these measures in cats, we manually contoured the brain, cranial box, and lateral ventricles from serial slices (Figs 2-4). Cranial box volume was derived by subtracting brain volume from total cranial volume. For comparison with humans, we used previously reported whole brain and cranial box volumes from the OASIS dataset (*n*=436; Marcus et al., 2007). Human lateral ventricle volumes were taken from the Brain Chart resource (Bethlehem et al., 2022). All volumetric measurements were quantified using ImageJ and OsiriX. We did not rely on templates because we used images of different resolutions.

### Brain necropsy reports

We found four cases reporting brain atrophy from cat necropsy reports from the Auburn College of Veterinary Medicine. These necropsies mentioned cerebrocortical atrophy, hippocampal atrophy, and widening of cortical sulci, suggesting that aged cats show brain atrophy. These mentions guided our analyses of *in vivo* MRI scans (Figs 2-4).

### Quantitative analyses of brain structures

We quantified brain metrics, such as gyrification and interthalamic adhesion, to characterize age-related changes in brain structure. We measured the interthalamic adhesion, which is a bridge of tissue that connects both thalami, and changes with brain atrophy in companion animals (Fig. 3; Noh et al., 2017; Blinkouskaya and Weickenmeier, 2021; Hasegawa et al., 2005). We used a coronal slice to measure the interthalamic adhesion thickness (where thickest). We measured the interthalamic adhesion area from the brain's midsagittal plane. We normalized the interthalamic adhesion thickness by dividing it by the brain height, which is the vertical distance from the most dorsal to ventral brain landmarks. We quantified gyrification on a single coronal slice by calculating the ratio of the perimeter that traced the gyri and sulci to the outer perimeter of the brain. Table S6 provides additional details of measurements.

### Brain metrics to align age

We fit a smooth spline to model the relationship between age and brain metrics separately for humans and cats. We identified the age at which brain metrics (e.g. interthalamic adhesion, brain volume) reach their maximum or its minimum before they decline or increase with age. If the metric decreased with age, we used the smooth spline to calculate the age at which the values fall to specific percentages (e.g. 90%, 80%, 70%; see Tables S1, S3). Because the MRI are from different sources and sexes, we fit smooth splines separately by sex and by group to evaluate whether the MRI type impacts brain measurements. Age-related variation in brain structure was very similar in males and females and by MRI type (Fig. 4; Fig. S3). We also found that the MRI data fell within 95% confidence intervals from other versions in cats and humans, which demonstrates that observations derived from MRI fall in line with other observations (Fig. S1).

### Comparison of MRI data with other observations

Since the cat brain MRIs were collected from different scanners, we compared observations from the 3T and 7T MR scanner (Fig. 4), and we tested whether observations from both the 3T and 7T MRI scanners fall in line with other observations (Fig. S1B). We fit a smooth spline to log-transformed observations (degrees of freedom=30) from cats and humans where observations from MRI were omitted (R^2^=0.975; *n*=230 time points; *n*=460 observations). We created 1000 new datasets by randomly sampling the dataset (omitting MRI data) with replacement. We then fit a new spline (with a smoothing parameter spar=0.9) to every bootstrapped sample. We use the standard deviation of the bootstrapped predictions to generate the 95% prediction intervals (Fig. S1B). Observations derived from MRI fall within the 95% confidence intervals of the bootstrapped data, which show the MRI data align with other observations (Fig. S1).

### Health records to equate ages across species

We used health records from multiple sources, including the Auburn Veterinary Clinic, the colony, Project CatAge, and the primate aging database (PAD). Many metrics are collected for health purposes, and these metrics vary with age. For example, ALP declines postnatally and subsequently becomes relatively invariant. These age-related patterns are conserved across different mammalian species (e.g. cats, chimpanzees, humans). Similarly, body weights increase postnatally, plateau, and subsequently decline later in life. We used the library R package easynls (e.g. model=3) to implement non-linear regressions to extrapolate corresponding ages across populations. We first fit a smooth spline through ALP versus age, and we extrapolated predicted values. We then used the R package easynls to test whether a piecewise linear model (y∼a+b×(x−c)×(x≤c)) accounts for a significant percentage of the variance. We extrapolate the age the plateau is reached (Tables S1, S4; Dataset 3). We also quantified when the values reached a percentage of the plateau (e.g. 120% of the plateau). We did not find significant differences in the pace of development between males and females. The slope of a linear model fit to health-related metrics (F=239.8, d.f.=59, adj R^2^=0.80; *P*<0.01, *n*=61) was not significantly different from 1 (slope=0.92; t value=−1.41; *P*=0.16; Fig. 7D). We use these as observations in the model used to equate ages across species.

### Blood chemistry profiles from the colony and Auburn CVM

Blood chemistry profiles from domestic shorthairs in the colony were collected from 2016 through 2021. A board-certified veterinary clinical pathologist re-evaluated health metrics collected from SRRC colony cats to confirm that the cats were healthy (*n*=99 samples; *n*=52 individuals; min-max: 0.12-9.9 years after birth; mean: 2.6 years after birth). We used blood chemistry profiles from apparently healthy cats collected from Auburn clinic from 2015 to 2023 (e.g. *n*=224 ALP observations; 197 cats; 85 females and 84 males; age min-max: 0.39-17.82 years after birth; mean: 8 years after birth). Most of these chemistry profiles are from domestic shorthairs, a few mixed breeds, some domestic longhair cats, and a few specialty breeds (e.g. Siamese cats, *n*=5; Ragdoll cats, *n*=3). These health metrics were not collected for the purposes of this study, which obviates the need for IACUC approval. Information about the cat's owner was removed before we gained data access.

### Project CatAge blood work

We asked cat owners to provide information about their cat's health records and life history (e.g. age, indoor or outdoor) on social media. We included cats in our analysis if their owners were confident of their cat's age or if they obtained them as kittens (i.e. less than 8 weeks of age). Project CatAge was IRB exempt by Auburn University. The health of these cats had been re-evaluated under the guidance of a board-certified veterinarian (see supplementary information). We included animals' blood values found within the normal range supplied by each health report. We included elevated ALP and phosphorus in cats under 1 year of age because these values are typically high in kittens (Levy et al., 2006; Pineda et al., 2013). We also included ALP below the normal range because it is not considered clinically significant (Oikonomidis and Milne, 2023). We excluded blood work from cats with dehydration (Ford and Mazzaferro, 2011), hyperthyroidism (Vaske et al., 2016), chronic kidney disease (Vervloet et al., 2017), bladder inflammation (Vaden et al., 2004), those receiving steroids (Ginel et al., 2002) and non-steroidal anti-inflammatory drugs (Hadjicharalambous et al., 2021) because these conditions and medications may impact blood chemistry profiles. We retained blood values for cats before they developed chronic kidney disease and hyperthyroidism (Syme et al., 2002). Cats with chronic localized conditions (e.g. megacolon, dental disease, osteoarthritis) were included in our dataset if there was no evidence of systemic inflammation or changes to blood parameters. Some tests do not include all metrics of interest. We excluded 13 blood tests from ten cats, and we excluded ALP values from two cats for health reasons. We collected 91 blood tests (i.e. total protein, ALP, creatinine, phosphorus) from 47 spayed or neutered cats. We used 78 blood tests from 45 cats (22 males; 23 females; ages 0.34 years old to 18.12 years old). Most of the cats were domestic shorthairs, lived indoors, and were from Alabama.

### Age-related pathologies

We collated information on age of diseases onset and progression, including observations from age-related changes in the prevalence of cataracts, and the minimum age of reported Alzheimer-related neuropathologies (e.g. brain amyloid, tangles; de Sousa et al., 2025; Table S1) for humans, chimpanzees, and cats (de Sousa et al., 2025). We excluded individuals with neurological conditions associated with early AD pathology (e.g. Down syndrome; see Gunn-Moore et al., 2007; Davidson et al., 2018), but we included data from individuals with late-onset seizures, defined as occurring after 5 years of age in cats and after 60 in humans (Hazenfratz and Taylor, 2018; Cleary et al., 2004), because AD-related pathology have links to seizures (Sordo et al., 2021).

### The translating time model

The dataset contains missing data (Fig. 1G; Table S1). We used the library package Amelia, which is an R library package designed for imputation of incomplete multivariate datasets, to generate imputed datasets (*n*=10; Zhang, 2016). These imputed values could not exceed the maximum reported lifespan for the relevant species. We selected the dataset generating the highest minimum correlation across individuals. We then denoised the imputed dataset with a principal component analysis. We used the imputed dataset to calculate an event scale (Fig. 1F), which is an ordering of time points averaged across species (Fig. 8). We produced a weighted average in the event scale, which is inversely proportional to the amount of missing data per species (Fig. 1G). The event scale is calculated by subtracting each averaged time point by the minimum time point and dividing that value by the difference between the maximum and minimum time point. Accordingly, the event scale ranges from 0 to 1. We fit a linear model with a natural spline to accommodate selective accelerations and decelerations in the pace of development and aging in some species.

### Accuracy of the model's predicted values

We generated a model to predict ages across multiple species, and we evaluate the accuracy of this model by comparing predicted values from the model with unimputed observations (Fig. S2). We fit a smooth spline to unimputed observations and tested whether predicted values from the linear model fall in line with observations in humans and cats (Fig. S2). We fit a smooth spline to log-transformed years post-conception (R^2^=0.978; *n*=248 time points; *n*=496 observations; spar=0.9), and we used 1000 bootstrap iterations to generate 95% prediction intervals. Our model's predictions generally align with observations across different ages, though a slight discrepancy exists between 0.5 and 1 year of age in cats and their correspondence in humans (Fig. S2). Given the strong concordance between observations and the model's predicted ages, we use the model's output (Fig. 8) and unimputed observations (Fig. 7) interchangeably throughout the text.

## Supplementary Material



10.1242/biolopen.062604_sup1 Supplementary information

Table S1. Timepoints and observations in cats, humans, mice, and chimpanzees. These data are used to translate ages across species. Abbreviations: PCD: post-conception days. Statistics may be 1 (minimum), 2, (mean, median, mode, or unknown) or 3 (maximum). If statistics were unknown, we set it the statistics to 2. In the column denoting sex, the “Male+Female” designation is used for pooled data containing both sexes, and for instances where the sex of the subjects was not specifically recorded.

Table S2. Timepoints for cats, humans, mice and chimpanzees.

Table S3. Brain metrics data from colony and research cats. Age is expressed in years post-birth.

Table S4. Blood work data in colony cats. Age is expressed in years post-birth. Phosphorus is shown in milligrams per deciliter, alkaline phosphatase is in units/liter, and creatinine is in milligrams per deciliter.

Dataset 1. This R script works with Table S1 to translate ages across species.

Dataset 2. This R script works with Table S3 to extract observations from age-related variation in blood chemistry profiles.

Dataset 3. This R script works with Table S1 to compare the pace of development between breed and non-breed cats.

Dataset 4. This R script works with Table S1 to compares the pace of development between wild and domesticated cats.

Dataset 5.

Dataset 6.

## Supplementary material

Supplementary tables (Tables S1-S4) include the translate time dataset, blood work, and MRI brain metrics used to generate age equivalencies across species. Tables S1 and S2 house time points from age-related brain variation and health metrics. Datasets 1-6 contain multiple R scripts and data used to generate age alignments, extract age of observations from blood work, and to compare the pace of development across feline species.
