# Supplementary material for: Cat brains age like humans: translating time shows pet cats live to be natural models for human aging
Source: Biol Open. 2026 Jun 22;15(6):bio062604. doi: 10.1242/bio.062604 (PMC13382973; doi:10.1242/bio.062604)
Supplement: Supplementary information [file biolopen-15-062604-s1.pdf]

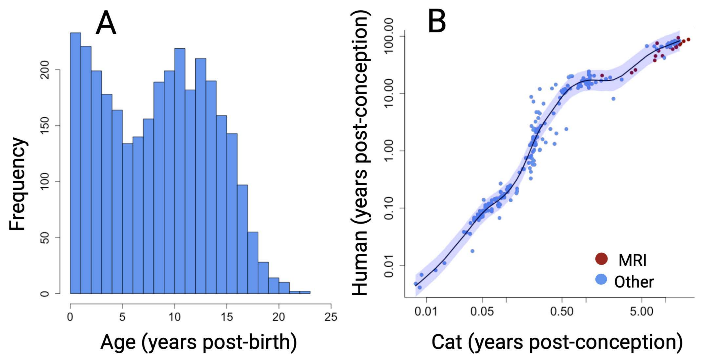

Fig. S1. (A) Histogram of age of cats visiting the Auburn clinic. Cats visit the clinic in their teens, but there is a sharp fall in the number of cats visiting the clinic after 12 years of age, which makes sampling aged cats a challenge. For cats with multiple visits, we recorded the age at the last consultation. (B) The age of observations taken from MRI data are in red and other time points are in blue. The 95% prediction intervals span the 2.5th and 97.5th percentiles of the bootstrap predictions.

Biology Open • Supplementary information

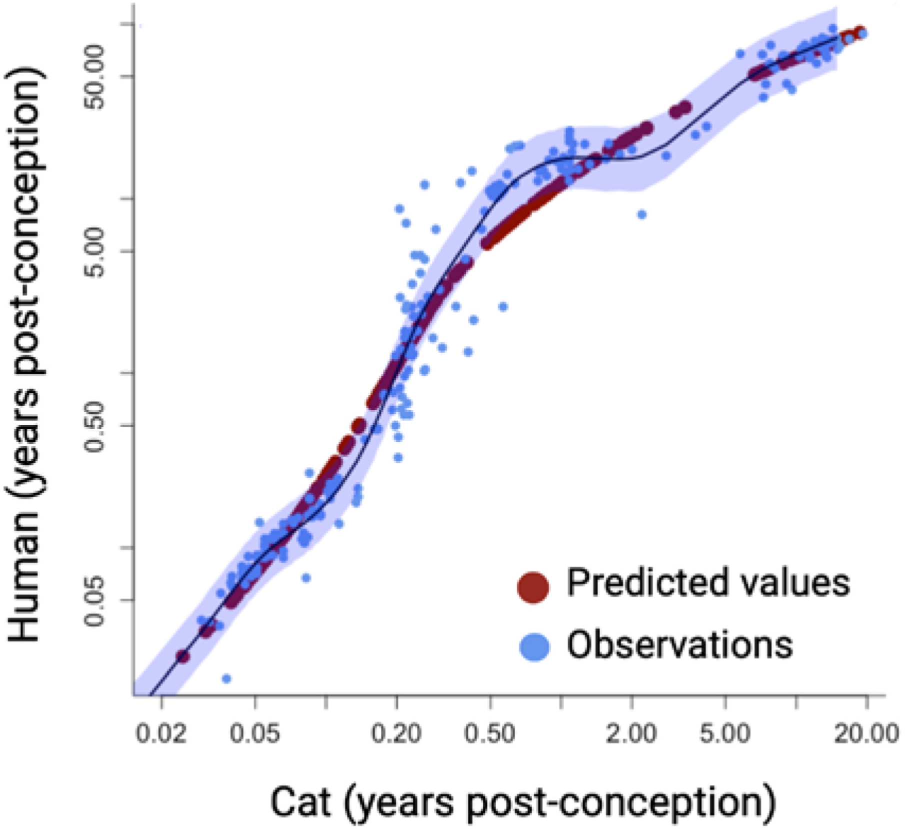

Fig. S2. (A) Scatterplot of unimputed observations compared with predicted values from the translating time model in cats and humans. We compared predicted values with unimputed observations. Predicted values fall within 95% prediction intervals. Given these observations, we use unimputed observations and predicted values interchangeably in the text.

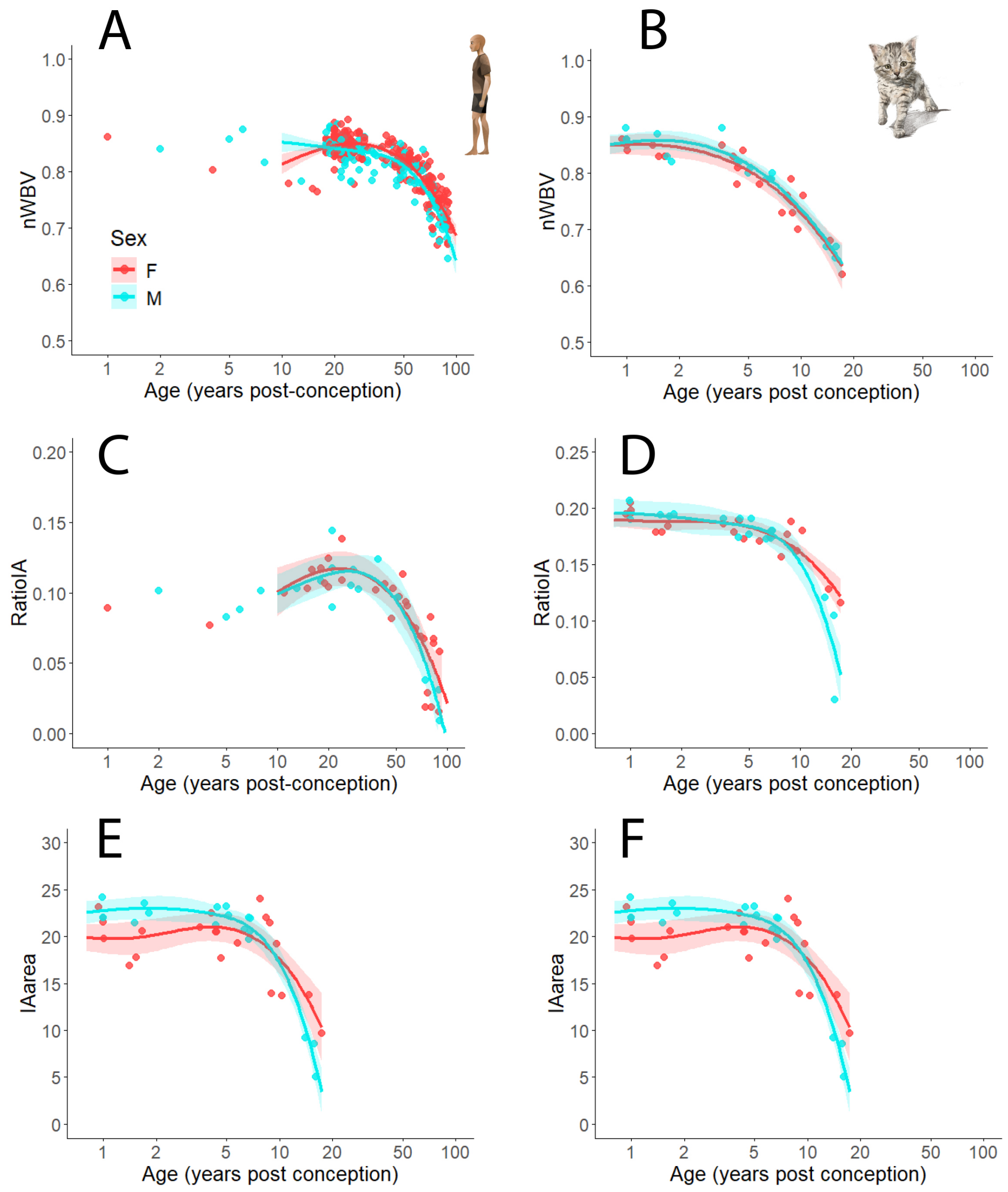

Fig. S3. We fit smooth splines and confidence intervals separately for males and females to assess whether there are sex differences in age-related patterns of brain change. Normalized brain volume in humans (A) and cats (B) decrease with age. There is little variation between males and females. The normalized interthalamic adhesion thickness (C, D) and area (E, F) decline with age. There is extensive overlap in normalized brain volume in males and females. Males appear to show accelerated reductions in interthalamic adhesion area and thickness after 10 years old in cats and after 50 years old in humans.

Table S1. Timepoints and observations in cats, humans, mice, and chimpanzees. These data are used to translate ages across species. Abbreviations: PCD: post-conception days. Statistics may be 1 (minimum), 2, (mean, median, mode, or unknown) or 3 (maximum). If statistics were unknown, we set it the statistics to 2. In the column denoting sex, the "Male+Female" designation is used for pooled data containing both sexes, and for instances where the sex of the subjects was not specifically recorded.

Available for download at
https://journals.biologists.com/bio/article-lookup/doi/10.1242/bio.062604#supplementary-data

Table S2. Timepoints for cats, humans, mice and chimpanzees.

Available for download at
https://journals.biologists.com/bio/article-lookup/doi/10.1242/bio.062604#supplementary-data

Table S3. Brain metrics data from colony and research cats. Age is expressed in years post-birth.

Available for download at
https://journals.biologists.com/bio/article-lookup/doi/10.1242/bio.062604#supplementary-data

Table S4. Blood work data in colony cats. Age is expressed in years post-birth. Phosphorus is shown in milligrams per deciliter, alkaline phosphatase is in units/liter, and creatinine is in milligrams per deciliter.

Available for download at
https://journals.biologists.com/bio/article-lookup/doi/10.1242/bio.062604#supplementary-data

Table S5. Summary statistics from smooth splines fit to brain metrics versus age in cats and humans.

Metric	R^2	Adjusted R^2	p-value	Species
Interthalamic adhesion ratio	0.7701	0.7492	1.615e-13	Cat
Interthalamic adhesion area	0.7111	0.6842	4.152e-11	Cat
Normalized brain volume	0.8391	0.8245	< 2.2e-16	Cat
Subarachnoid volume	0.8404	0.8259	< 2.2e-16	Cat
Lateral Ventricle Volume	0.4939	0.4479	3.697e-06	Cat
Gyrus depth	0.5073	0.4615	2.926e-06	Cat
Interthalamic adhesion ratio	0.757	0.7339	2.116e-12	Human
Interthalamic adhesion area	0.8155	0.7979	6.973e-15	Human
Normalized brain volume	0.7488	0.7458	< 2.2e-16	Human
Subarachnoid volume	0.5282	0.5227	< 2.2e-16	Human
Lateral Ventricle Volume	0.9984	0.9983	< 2.2e-16	Human
Gyrus depth	0.755	0.7316	2.516e-12	Human

Biology Open • Supplementary information

Table S6. Description of brain measurements.

Brain Structure	Description
**Brain volume**	is measured by contouring the brain manually or semi automatically from multiple consecutive slices.
**Total Intracranial box volume**	is measured by contouring the intracranial bones manually or semi automatically from multiple consecutive slices. This includes ventricles.
**Subarachnoid volume**	is calculated by subtracting the brain volume from the total intracranial box volume.
**Lateral ventricles' volumes**	are contoured separately from multiple slices.
**Normalized Whole Brain Volume**	is the ratio of the brain volume (excluding the lateral ventricles volume) relative to the total intracranial box volume. We used these normalization procedures to account for variation in head size.
**Interthalamic adhesion thickness**	We used a coronal slice to measure the bridge of tissue that connects both thalami. We measured the interthalamic adhesion where it was thickest.
**Brain height**	Brain height is defined as the vertical distance from the highest dorsal point to the most ventral point measured on the same slice where interthalamic adhesion thickness measurements were made.
**Ratio of the interthalamic adhesion thickness to brain height**	We normalized the interthalamic adhesion thickness to the brain's height.
**Interthalamic adhesion area**	The interthalamic adhesion area is measured from a mid-sagittal slice where the thalami are the smallest.
**Gyrification**	Gyrification was quantified as the ratio of the perimeter tracing the gyri and sulci to the perimeter tracing the outer surface of the brain. Both perimeters were measured from the same coronal slice used to assess the thickness of the interthalamic adhesion.

Dataset 1. This R script works with Table S1 to translate ages across species.

Available for download at
https://journals.biologists.com/bio/article-lookup/doi/10.1242/bio.062604#supplementary-data

Dataset 2. This R script works with Table S3 to extract observations from age-related variation in blood chemistry profiles.

Available for download at
https://journals.biologists.com/bio/article-lookup/doi/10.1242/bio.062604#supplementary-data

Dataset 3. This R script works with Table S1 to compare the pace of development between breed and non-breed cats.

Available for download at
https://journals.biologists.com/bio/article-lookup/doi/10.1242/bio.062604#supplementary-data

Dataset 4. This R script works with Table S1 to compares the pace of development between wild and domesticated cats.

Available for download at
https://journals.biologists.com/bio/article-lookup/doi/10.1242/bio.062604#supplementary-data

Dataset 5.

Available for download at
https://journals.biologists.com/bio/article-lookup/doi/10.1242/bio.062604#supplementary-data

Dataset 6.

Available for download at
https://journals.biologists.com/bio/article-lookup/doi/10.1242/bio.062604#supplementary-data
