# Supplementary material for: Cat brains age like humans: translating time shows pet cats live to be natural models for human aging
Source: Biol Open. 2026 Jun 22;15(6):bio062604. doi: 10.1242/bio.062604 (PMC13382973; doi:10.1242/bio.062604)
Supplement: Supplementary file 1 [file biolopen-15-062604_review_history.pdf]

Cat brains age like humans: Translating Time shows pet cats live to be natural models for human aging

Capucine Januel, Elijah Morrow, Ryan Gibson, Amanda Gross, Alexandra A. de Sousa, Brier A. Rigby Dames and Christine Charvet
10.1242/bio.062604

Editor: Sandhya Koushika

Review timeline

Original submission:	4 April 2026
Editorial decision:	15 April 2026
First revision received:	30 May 2026
Accepted:	7 May 2026

Original submission

First decision letter

MS ID#: bio.062604

MS Title: Cat brains age like humans: Translating Time shows pet cats live to be natural models for human aging

Authors: Christine Charvet, Capucine Januel, Elijah Morrow, Ryan Gibson, Amanda Gross, Alexandra A. de Sousa and Brier A. Rigby Dames

I have now reached a decision on the above manuscript.

The reviewer reports are shown at the bottom of this email.

As you will see, the reviewers raised a number of substantial criticisms that prevent me from accepting the paper at this stage.

They suggest, however, that a revised version might prove acceptable, if you can address their concerns. If you think that you can deal satisfactorily with the criticisms on revision, I would be pleased to see a revised manuscript. We would then return it to the reviewers.

At this stage, we also ask you to ensure your manuscript complies with our formatting guidelines. Provided you are able to fully address the referees' comments, we are positive about publication of your paper (we accept over 95% of revision submissions) and therefore hope you won't mind any extra work involved in reformatting your manuscript at this point.

Please upload both a 'clean' version of your Word file, along with a highlighted version clearly showing where you have made changes in the revised manuscript. Please avoid using 'Track changes' in Word files as these are lost in PDF conversion.

I should be grateful if you would also provide a point-by-point response detailing how you have dealt with the points raised by the reviewers in the 'Response to Reviewers' box. Please attend to all of the reviewers' comments. If you do not agree with any of their criticisms or suggestions please explain clearly why this is so.

© 2026. Published by The Company of Biologists under the terms of the Creative Commons Attribution License (https://creativecommons.org/licenses/by/4.0/).

Reviewer 1

Comments for the author

Thank you for inviting me to review this manuscript. My overall judgement is that the paper contains an interesting comparative dataset and a potentially useful descriptive framework, but the translational claim is currently stronger than the evidence supports. For this reason, it is my view that this paper is potentially publishable after substantial revision. The core problem is that the manuscript shifts too quickly from age-alignment and gross morphometry (which are largely descriptive) to the claim that pet cats are "natural models" of human neurodegeneration. From my perspective, the evidence presented supports age-related structural similarity and lifespan alignment far better than it supports translational disease modelling.

Experimental quality

This is an innovative, observational, integrative modelling study built from heterogeneous datasets that include cat MRI, human MRI, blood chemistry, developmental milestones and previously published cross-species datasets. This is absolutely fine in principle, but it does mean that the standard for confound control, data harmonisation and (critically) restraint in inference should be MUCH higher than it currently is.

Relating to the use of proper controls, the paper does include some reasonable comparators. Human MRI cases are restricted to CDR=0, and clinical cats with overt brain lesions were excluded by a veterinary neurologist. The manuscript also compares clinic and colony cats, and breed/non-breed and domestic/wildcat developmental trajectories, which are all legitimate internal comparators. However, the controls are not sufficient for the strength of the causal or translational argument being presented. The cat MRI data are drawn from two very different acquisition environments, using different scanners, coils, pulse sequences, resolutions, and anaesthetic regimens. The authors acknowledge this heterogeneity and say they compared groups to assess scanner effects, but they do not appear to model scanner/platform as a covariate, and they do not show a formal harmonisation analysis. Since the key claims they are making rely on relatively subtle morphometric trajectories, scanner and sequence heterogeneity are potentially serious confounds and cannot be ignored as noise.

Relatedly, the morphometry is carried out manually, but there is no statement about blinding, intra-rater reliability, inter-rater reliability, or segmentation validation. For a study in which the central measurements depend on manual delineation across heterogeneous image qualities, that omission is alarming to say the least.

The statistical approach is partly appropriate but also partly under-justified. Smooth splines are fine non-linear lifespan trajectories. Linear models comparing matched developmental events and tests of slope against 1 are also fine. The Kolmogorov-Smirnov test for age-distribution differences and Games-Howell post hoc testing are acceptable in the comparisons shown. But the inferential framework is not good. The manuscript relies heavily on extracted spline landmarks such as peak values and percentages of plateaus/maxima, yet there is very little discussion of uncertainty propagation from the spline fits into the age-translation model. Similarly, the paper says missing observations were imputed, but the main text does not make the imputation strategy (inlcuding assumptions used or sensitivity analysis) sufficiently explicit for a claim this broad. Figure 1 explicitly states that data were imputed when unavailable and that missingness differs by species; that raises the bar for robustness checks, which are not evident in the main manuscript (unless I missed it).

Reproducibility

For the MRI component, the biological replicate numbers are modest for the cats: 53 cats total, split into 18 clinic and 34 colony animals, vs. 446 humans. I am not saying that this is completely inadequate for a descriptive ageing study, but once the data are further subdivided by source and used to infer lifespan trajectories, the cat sample becomes fairly thin, especially at the oldest ages where the strongest claims are made. The visual examples of extreme atrophy are also anecdotal exemplars, not population-level evidence on their own. For the blood chemistry comparisons, the n

© 2026. Published by The Company of Biologists under the terms of the Creative Commons Attribution License (https://creativecommons.org/licenses/by/4.0/).

values are more respectable: AU clinic n=169, Project CatAge n=45, colony n=99 for the ALP-based age comparison. That part is better powered. But those data address age distribution and developmental pace more than brain ageing per se.

Completeness

The narrow conclusion that cats and humans show broadly similar age-related changes in several gross brain metrics is supported reasonably well by the data presented. The paper also supports the claim that pet cats seen clinically tend to be older than colony cats, and therefore provide access to older feline ages than colonies typically do. But, the broad conclusion that this work "establishes cats as powerful and naturally occurring models for the study of neurodegeneration" is not supported by the data shown. The study excludes overt lesions and tries to exclude cognitive dysfunction, but the cats are not phenotyped for longitudinal cognitive decline, neuropathological burden, or mechanistic similarity to human neurodegenerative disease. In fact, the authors explicitly concede that they cannot rule out cognitive dysfunction in some cats. That in itself seems to weaken the claim that the MRI changes clearly represent normal ageing rather than mixed ageing-plus-disease, and it certainly prevents a strong inference about modelling neurodegeneration.

There are also design issues that limit interpretation. First, the pet-versus-colony comparison is confounded by age distribution itself. The manuscript's conclusion is that pet cats show more atrophy largely because they are older, not because pet status per se changes brain ageing. That means the paper cannot distinguish environment, veterinary ascertainment, scanner differences, and ageing exposure cleanly. Second, the translational model is partly built on imputed missing observations across species. Without clear sensitivity analyses, how do we know how much the headline alignments depend on the imputation procedure rather than the observed data? Third, the study uses cross-sectional rather than longitudinal imaging. That is ok for initial trajectory fitting, but it means statements about within-animal ageing or rates of decline should be kept cautious. Fourth, the manuscript uses age-related blood chemistry and developmental milestones to support cross-species age alignment. That may be acceptable for a general framework, but those measures do not directly validate the cat as a model of human brain ageing or neurodegeneration.

Finally, there are certainly missing experiments that could disprove the conclusion, in my view. The most important would be a longitudinal cognitive phenotyping linked to MRI in ageing cats with neuropathological confirmation in aged cats spanning cognitively normal and impaired animals, formal harmonisation or replication of MRI-derived morphometry in a scanner-controlled subset, and a clear demonstration that the cross-species mapping is robust to alternative model specifications and to removal of imputed data. None of these would be trivial, and the authors do acknowledge one limitation, namely that some cats may still have cognitive dysfunction despite screening. But they do not, in my view, adequately discuss the broader limitations of heterogeneity, imputation, and disease-versus-normal-ageing inference.

Scholarship

The only sense in which the scholarship is weak is in discussing evidence that argues against the stronger translational conclusion. The paper does not seriously engage with the distinction between resemblance in gross structural ageing versus validated disease homology. It also does not adequately discuss literature on the limits of opportunistic clinical imaging cohorts, ascertainment bias in veterinary populations, or the confounding impact of mixed scanner/protocol data on morphometry. I also think that the advocacy, especially around One Health and the inadequacy of laboratory models, is creeping into over-reach given the presented evidence.

<u>Reviewer 2</u>

Comments for the author

The authors use a series of brain metrics from cats and humans, other health-related metrics from cats and humans, and other aging data from chimpanzees and mice, which were obtained from various sources (apparently from this and previous studies) to establish aligned developmental and aging trajectories across human and cat lifespans and propose that cats, most clearly pet cats in

© 2026. Published by The Company of Biologists under the terms of the Creative Commons Attribution License (https://creativecommons.org/licenses/by/4.0/).

clinics could be a powerful model for the study of human aging, most notably brain aging. However, the way this potentially interesting article is written is not sufficiently clear and is non-linear, making it very difficult to follow and critically interpret. Figure 1, for instance, is presented at the very beginning, but the results of, say, Panel A (Figure 1A) are mostly described/dissected in the last sections of the Results. This and other methodology and data presentation issues described below makes reading the manuscript very confusing and time-consuming. Throughout the manuscript, it is hard to understand what corresponds to new data collected for the first time in this study, data extracted from previous publications, data computationally digitized from graphs in previous studies, and data that were imputed. How the different metric data were integrated is also not entirely clear. These points must be clarified to allow a full critical evaluation of experimental quality, sample quality and size, reproducibility, and the relevance and novelty of the analyses. In the current format, it is not possible to evaluate how well the title and abstract reflect the data and how well the authors discuss the merits of their data, without making assumptions or deductions. For instance, what data were used to obtain the graphs in Figure 1C? Figure 1C is not directly referred to in the text, nor are Figures 1B or 1D, for instance. I did not check the other figures.

Abstract (and throughout the text)

- Some terms are poorly defined or used in loose ways. For instance, colony and pet cats. Abstract, line 31: "pet and colony cats", then in line 35 it mentions cats from clinics, zoos, and colonies. Please use precise nomenclature and/or explain what categories are equivalent.

- Please clarify precisely what "colony cats" means; it is not clear to the average non-specialist reader. In the methods, please define "colony". Define what is the Auburn colony and Japan colony, and when "colony" is used alone in the text, please state what does it refer to.

- Specify whether the pet cats come from clinics only and/or zoos or elsewhere.

- In Figure 5, a new class appears: "clinics vs research". Please clarify what "research" means and ensure consistency throughout.

Figures and figure legends

If possible, please provide more detail in the figures or figure legends about the data (n; whether values are imputed or observed); plots (box plots (and whiskers) and line plots—e.g., what the lines and shaded regions represent (95% confidence intervals?)); and basic statistics for the fits (as approximately done for Panel D in Figure 6; although the box plot box and whiskers do not seem to be defined in that panel or in the methods).

- Page 7, line 135: "structures increase with age (e.g., interthalamic adhesion)…" Please verify. As interthalamic adhesion ultimately decreases with age, perhaps the authors meant something like "subarachnoid volume", which increases with age in both cats and humans?
- Lines 136-137: A word may be missing in "… time points in human and ??? (Figure 4)." Perhaps "cats"?
- There are double punctuation marks after e.g., or i.e., in lines 132, 137, and 138. Please check these and verify elsewhere in the manuscript.

Results

- Results, page 7 (quoted text):
"We used these smooth splines to extract corresponding time points in humans and (Figure 4). For example, we extracted the age at which the brain volume is maximal (i.e.., 100%) and when it reaches a percentage of its maximum size (e.g.., 90%, 80%) in the two species. We did this for multiple metrics to extract corresponding time points between humans and cats."
It is unclear how the corresponding time points from multiple metrics were integrated across metrics. Please clarify.
- Results, Figure 5: Please define ITA in the legend. Please provide statistical information (what the * indicates), describe the box plot (box and whiskers), and provide N. The relevant information appears to be in the text (consider moving it to the legend).

© 2026. Published by The Company of Biologists under the terms of the Creative Commons Attribution License (https://creativecommons.org/licenses/by/4.0/).

- Results, page 11, lines 185-190: The phrase "multiple sources" is not sufficiently precise to allow replication. Please specify the sources used (and verify this throughout the manuscript).

- Results, Figure 6:
- Panel A: Consider capitalising "A" in "Catage". I also suggest using a consistent font size across all figures to improve readability, if possible.
- Panel C: Please check "phosphotase", and note that the inset is barely readable.
- Panel E: Please check the y-axis label. Maybe include it, even if redundant with panel D.

Materials and Methods

- Lines 353-354: Please specify (at least in Table S1) exactly which data were extracted using WebPlotDigitizer v4 (Rohatgi, 2025), and the conditions used (e.g., manual vs automated extraction).

- Line 355: Typo: "we discussed howe..."

- Line 501:
"The dataset contains missing data (Figure 1E; Table S1). We used the library package Amelia to generate imputed datasets (n=10)."
It is unclear what data are missing, which missing values were imputed, and in which datasets. The authors refer to Table S1, but it is unclear what is missing in Table S1. What is the relationship between missing data and "unknown" in Table S1? What was done when a statistic was "unknown"? What the authors did with these data is very hard to follow. Please provide more information. Please also provide information on what the library package Amelia is. If it is an R package, please state so and, if possible, provide reference(s) for the packages.

Supplementary materials

- Table S1: Please include "This study" if/where the data is reported for the first time in this study, or describe from where it was extracted with a reference.

- Table S1 Legend: Missing a ")" at the end of the sentence. Please describe the "sex" categories—specifically, what does "male+female" mean?

- Table S3 Legend: Please provide units for all variables measured, where applicable.

- Table S4 Legend: Missing "y" in "Summar".

- Please include a discussion of the effects of imputed data on the interpretations made. Please specify exactly which data were imputed in each section that uses or mentions imputed data.

Reviewer's Responses to Questions
Experimental quality

Does each figure have the proper controls?
If 'No', please indicate reasons in Comments for Author box below.
Reviewer #1:
- No

Reviewer #2:
- No

Were the data analyzed using appropriate statistical tests?
If 'No', please indicate reasons in Comments for Author box below.
Reviewer #1:
- No

© 2026. Published by The Company of Biologists under the terms of the Creative Commons Attribution License (https://creativecommons.org/licenses/by/4.0/).

Reviewer #2:

- Yes

Reproducibility
Were experiments performed using adequate number of biological replicates?
If 'No', please indicate reasons in Comments for Author box below.
Reviewer #1:

- No

Reviewer #2:

- No

Does the methods section provide sufficient detail to permit reproducibility?
If 'No', please indicate reasons in Comments for Author box below.
Reviewer #1:

- No

Reviewer #2:

- No

Completeness
Are the manuscript's conclusions supported by the data?
If 'No', please indicate reasons in Comments for Author box below.
Reviewer #1:

- No

Reviewer #2:

- No

Scholarship
Do the authors cite and discuss the merits of data that would argue for and against their conclusion?
If 'No', please indicate reasons in Comments for Author box below.
Reviewer #1:

- Yes

Reviewer #2:

- No

Does the manuscript title & abstract accurately reflect the contents of the manuscript, without hyperbole?
If 'No', please indicate reasons in Comments for Author box below.
Reviewer #1:

- Yes

Reviewer #2:

- No

First revision

© 2026. Published by The Company of Biologists under the terms of the Creative Commons Attribution License (https://creativecommons.org/licenses/by/4.0/).

Author response to reviewers' comments

Dear Dr. Koushika

Thank you for your comments and the opportunity to respond to reviewers. We have made many of the suggested modifications. We outline these changes below, and we consider these changes have greatly improved the manuscript. We also edited the manuscript for clarity, moved text from the supplementary material to the main text and formatted the manuscript for *Biology Open*. We hope these changes are satisfactory for publication in *Biology Open*.

Comments from the Reviewers:

Reviewer 1: Thank you for inviting me to review this manuscript. My overall judgement is that the paper contains an interesting comparative dataset and a potentially useful descriptive framework, but the translational claim is currently stronger than the evidence supports. For this reason, it is my view that this paper is potentially publishable after substantial revision. The core problem is that the manuscript shifts too quickly from age-alignment and gross morphometry (which are largely descriptive) to the claim that pet cats are "natural models" of human neurodegeneration. From my perspective, the evidence presented supports age-related structural similarity and lifespan alignment far better than it supports translational disease modelling.

Thank you for your positive and critical feedback.

We agree that the original manuscript placed excessive emphasis on the concept of One Health. While we remain enthusiastic about the connection of our work to One Health, we recognize these topics fall outside the scope of this study. We have revised the abstract, introduction, discussion, and supplementary materials to prioritize the immediate implications of age translations and showcase validation of our models. These revisions, along with the specific points addressed below, ensure the paper remains focused on our core findings. We followed recommendations from both reviewers in making the following changes.

Experimental quality

This is an innovative, observational, integrative modelling study built from heterogeneous datasets that include cat MRI, human MRI, blood chemistry, developmental milestones and previously published cross-species datasets. This is absolutely fine in principle, but it does mean that the standard for confound control, data harmonisation and (critically) restraint in inference should be MUCH higher than it currently is.

Relating to the use of proper controls, the paper does include some reasonable comparators. Human MRI cases are restricted to CDR=0, and clinical cats with overt brain lesions were excluded by a veterinary neurologist. The manuscript also compares clinic and colony cats, and breed/non-breed and domestic/wildcat developmental trajectories, which are all legitimate internal comparators. However, the controls are not sufficient for the strength of the causal or translational argument being presented. The cat MRI data are drawn from two very different acquisition environments, using different scanners, coils, pulse sequences, resolutions, and anaesthetic regimens. The authors acknowledge this heterogeneity and say they compared groups to assess scanner effects, but they do not appear to model scanner/platform as a covariate, and they do not show a formal harmonisation analysis. Since the key claims they are making rely on relatively subtle morphometric trajectories, scanner and sequence heterogeneity are potentially serious confounds and cannot be ignored as noise.

Thank you for raising this point. Several of Reviewer 1's concerns focus on our use of multiple MRI scanners to track age-related brain changes. Before addressing our technical approach, we wish to clarify the scale of these data within the broader study.

The MRI data represents a very small fraction of our dataset. For example, the MRI data represent only 1.9% of timepoints in our dataset. There are only 18 MRI observations (9 time points) collected from MRI. We believe that similarities in brain atrophy patterns between the two species are compelling and worthy of discussion. However, there are few observations derived from MRI to

© 2026. Published by The Company of Biologists under the terms of the Creative Commons Attribution License (https://creativecommons.org/licenses/by/4.0/).

translate ages, which means that MRI type is unlikely to have a major impact on the study's primary findings.We applied multiple strategies to derive this conclusion. We still compare metrics from two different scanners in the manuscript (Figure 4). We also integrate a large number of observations (i.e., 938 time points) to generate age alignments across geriatric cats so as not to rely exclusively on MRI data. We now include an additional test to show that observations fall in line with other observations.

We tested whether observations derived from MRI data fall within the 95 % prediction intervals generated from all other observations (Fig. S1B). We fit a smooth spline to log-transformed observations (degrees of freedom=30) available in cats and humans (omitting observations derived from MRI data) and bootstrap the data to generate prediction intervals. As shown in Fig. S1B, the MRI observations (shown in red) fall within 95% prediction intervals generated from all other observations. These observations confirm that the MRI data are statistically consistent with other observations and that the MRI data do not skew cross-species age alignments.

We still include analyses to evaluate whether MRIs would confound our results. We compared brain metrics between colony and pet cats. We found that the two groups are nearly identical, with splines falling within 95% confidence intervals of one another (Figure 4). When controlled for age, the data from pet and colony cats are nearly identical. We contend that including scanner covariates would not change cross-species age alignments, especially since age-related brain atrophy is specific to the older pet cats rather than colony cats. Pet cats (but not colony cats) are studied in their teens. We consider the ability to model MR scanner co-variates to be questionable since we have no data for colony cats in their teens.

We consider that the deliberate inclusion of cats from varied environments constitutes a strength rather than a weakness in the study. Our age-translation models capture within species variation so that our cross-species age translations are applicable across feline populations.

We have now added the following in the Methods section and Figure S1B:

Comparison of observations from MRI data with other observations

Since the cat brain MRIs were collected from different scanners, we compared observations from the 3T and 7T MR scanner (Fig. 4), and we tested whether observations from both the 3T and 7T MRI scanners fall in line with other observations (Fig. S1B). We fit a smooth spline to log-transformed observations (degrees of freedom=30) from cats and humans where observations from MRI were omitted (R^2=0.975; n=230 time points; n=460 observations). We created 1,000 new datasets by randomly sampling the dataset (omitting MRI data) with replacement. We then fit a new spline (with a smoothing parameter spar = 0.9) to every bootstrapped sample. We use the standard deviation of the bootstrapped predictions to generate the 95 percent prediction intervals (Fig. S1B). Observations derived from MRI fall within the 95% confidence intervals of the bootstrapped data, which show the MRI data align with other observations (Fig. S1).

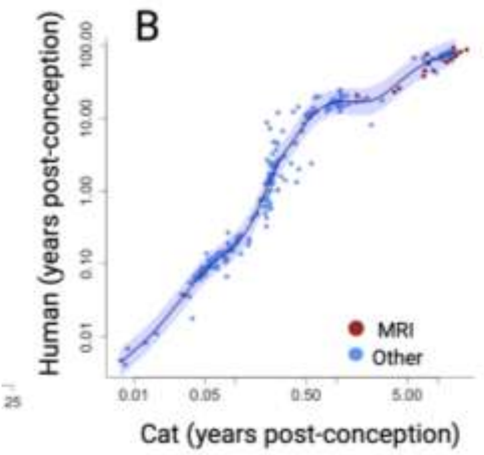

© 2026. Published by The Company of Biologists under the terms of the Creative Commons Attribution License (https://creativecommons.org/licenses/by/4.0/).

Figure S1. … (B) The age of observations taken from MRI data are in red and other time points are in blue.

We also reworked the Introduction to show the diverse metrics we use to translate ages. Previously, we focused on whether animals possess similar patterns of brain atrophy, which may have led the reader to believe we relied overwhelmingly on brain atrophy to generate cross-species age alignments. We should have introduced the different metrics we acquired to translate ages across the lifespan of different species. Figure 1C now includes a breakdown of the time points so that the reader can appreciate the MRI data accounts for a relatively small percentage of the time points we collected.

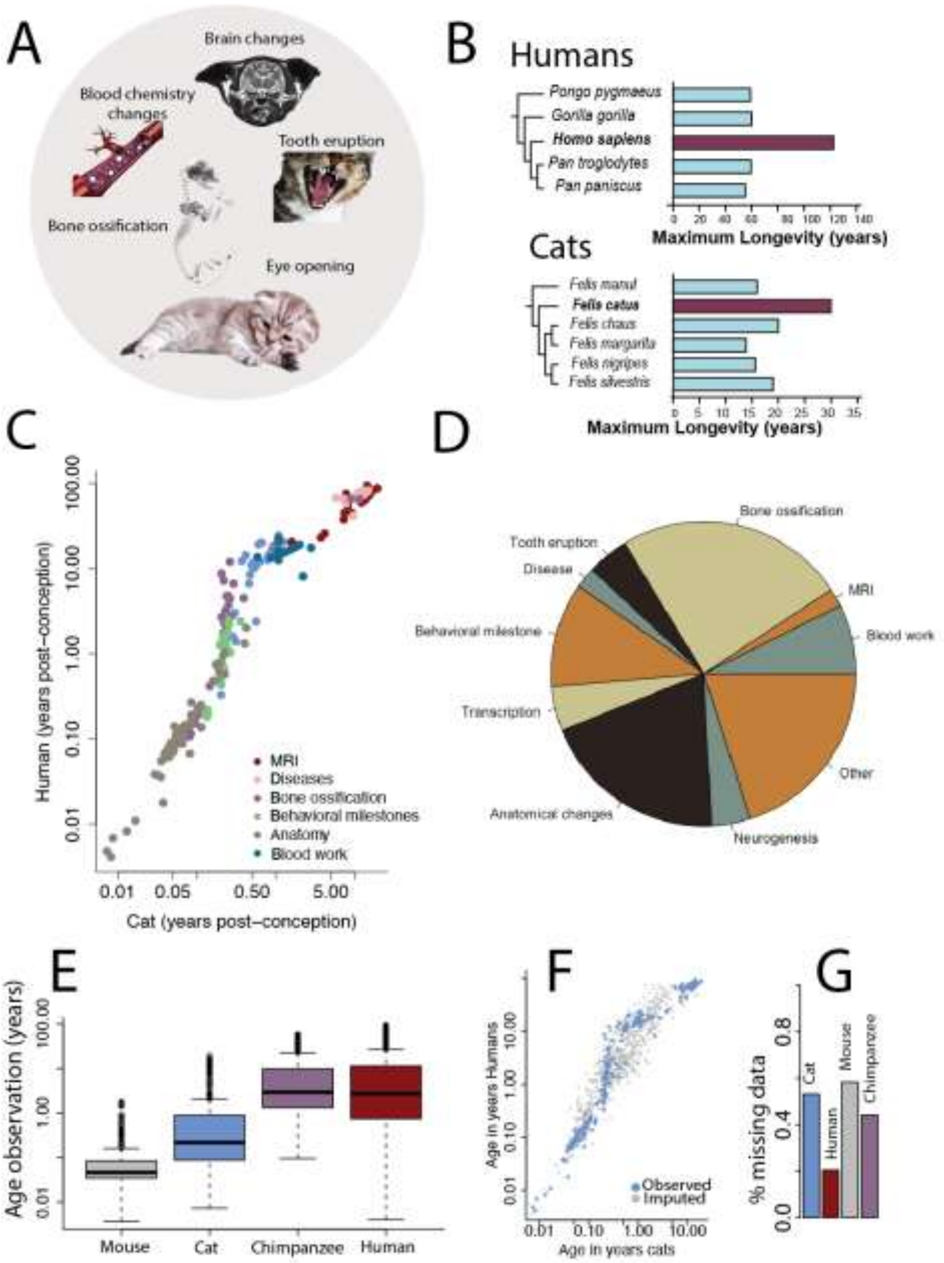

Figure 1 now focuses on the breakdown of the data in C and D. The reader can see the different metrics we collected. Figure 1D shows the MRI data accounts for a relatively small percentage of the time points we collected.

Relatedly, the morphometry is carried out manually, but there is no statement about blinding, intra-rater reliability, inter-rater reliability, or segmentation validation. For a study in which the

© 2026. Published by The Company of Biologists under the terms of the Creative Commons Attribution License (https://creativecommons.org/licenses/by/4.0/).

central measurements depend on manual delineation across heterogeneous image qualities, that omission is alarming to say the least.

While we acknowledge the reviewer's point, our primary objective was to translate ages between humans and cats, rather than compare pet and colony populations. Practically speaking, a 'blind' analysis is not feasible when comparing cat and human brains. Any researcher qualified to map feline brain structures should distinguish them from human samples. Without external funding, we are not ready to hire additional staff to replicate these tasks blindly. We did include images of MRIs to illustrate the big age-related structural changes we observed. It is also well known that the human brain atrophies with old age so we do not see a need to replicate analyses performed in humans.

The statistical approach is partly appropriate but also partly under-justified. Smooth splines are fine non-linear lifespan trajectories. Linear models comparing matched developmental events and tests of slope against 1 are also fine. The Kolmogorov-Smirnov test for age-distribution differences and Games-Howell post hoc testing are acceptable in the comparisons shown. But the inferential framework is not good. The manuscript relies heavily on extracted spline landmarks such as peak values and percentages of plateaus/maxima, yet there is very little discussion of uncertainty propagation from the spline fits into the age-translation model. Similarly, the paper says missing observations were imputed, but the main text does not make the imputation strategy (inlcuding assumptions used or sensitivity analysis) sufficiently explicit for a claim this broad. Figure 1 explicitly states that data were imputed when unavailable and that missingness differs by species; that raises the bar for robustness checks, which are not evident in the main manuscript (unless I missed it).

Thank you for your critical feedback, which is pushing us to improve our work. We have organized answers to these comments below.

Perceived issue with smooth splines: The reviewer makes reference to Kolmogorov-Smirnov test, which suggests that the smooth splines are in reference to the brain atrophy. Age-related changes in brain atrophy account for 1.9% of time points. It is true that the sample sizes we used to extract brain atrophy are small and are thus not amenable for bootstrapping analyses. The study of aged animals is challenging because there is a drop off in the number of individuals alive in old age. We now provide a distribution of cats visiting the clinic (Fig. 1A) to show that geriatric cats visiting the clinic are relatively few in numbers. Otherwise, readers may think that cats are readily available for study at any age.

Perceived issues with model fit: We utilized statistical models that capture a significant proportion of the variance. Specifically, we fit smooth splines to the interthalamic adhesion area, thickness and brain volume versus age because these account 68-99% of the variance in the MRI data, demonstrating these models account for a large percentage of the variance (Table S4). We consider we do not have sufficiently large sample sizes to bootstrap the MRI data. We do test whether the inclusion of MRI data significantly impacts results and we found that that was not the case (Figure S1B).

Perceived issue with imputation: We would like to clarify the role of imputation in our visualizations. The model presented in Figure 8 utilizes an event scale on the x-axis, which is derived from imputed data. However, observations in Figure 7 consist of unimputed observations. The full details of our imputation pipeline are documented in the scripts provided with the manuscript, though we recognize the importance of explicitly stating this distinction in the text and we have updated the text and figure legends accordingly. We refer to observations in figure 7 as unimputed observations throughout the manuscript. We also reiterate that observations are unimputed in the Materials and Methods section.

We have now included validation analysis comparing predicted ages from the model (based on the imputed event scale) against our unimputed observations (Figure S2B). As demonstrated in Figure S2B, the predicted values align closely with the observed data. This high degree of concordance justifies our interchangeable use of predicted and unimputed observations throughout the manuscript. We appreciate the suggestion to include this comparison, as we consider it

© 2026. Published by The Company of Biologists under the terms of the Creative Commons Attribution License (https://creativecommons.org/licenses/by/4.0/).

substantially strengthens the validity of our cross-species age alignments. We now include a section in the Materials and Methods section:

Accuracy of the model's predicted values

We generated a model to predict ages across multiple species, and we evaluate the accuracy of this model by comparing predicted values from the model with unimputed observations (Fig. S2). We fit a smooth spline to unimputed observations and tested whether predicted values from the linear model fall in line with observations in humans and cats (Fig. S2). We fit a smooth spline to log-transformed years post-conception (R^2=0.978; n=248 time points; n=496 observations; spar=0.9), and we used 1,000 bootstrap iterations to generate 95% prediction intervals. Our model's predictions generally align with observations across different ages, though a slight discrepancy exists between 0.5 and 1 year of age in cats and their correspondence in humans (Fig. S2). Given the strong concordance between observations and the model's predicted ages, we use the model's output (Fig. 8) and unimputed observations (Fig. 7) interchangeably throughout the text.

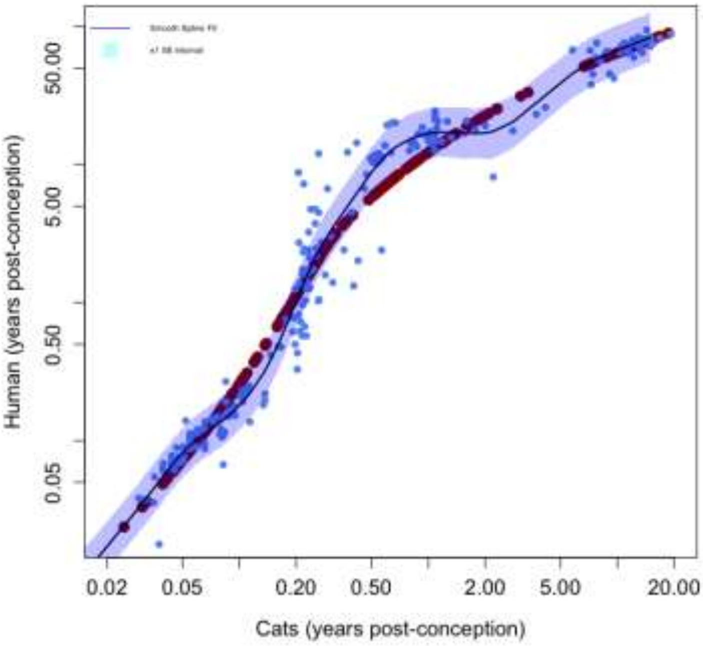

Figure S2. (A) Scatterplot of unimputed observations compared with predicted values from the translating time model in cats and humans. We compared predicted values with unimputed observations. Predicted values fall within 95% prediction intervals. Given these observations, we use unimputed observations and predicted values interchangeably in the text.

Reproducibility

For the MRI component, the biological replicate numbers are modest for the cats: 53 cats total, split into 18 clinic and 34 colony animals, vs. 446 humans. I am not saying that this is completely inadequate for a descriptive ageing study, but once the data are further subdivided by source and used to infer lifespan trajectories, the cat sample becomes fairly thin, especially at the oldest ages where the strongest claims are made. The visual examples of extreme atrophy are also anecdotal exemplars, not population-level evidence on their own. For the blood chemistry comparisons, the n values are more respectable: AU clinic n=169, Project CatAge n=45, colony n=99 for the ALP-based age comparison. That part is better powered. But those data address age distribution and developmental pace more than brain ageing per se.

We appreciate the opportunity to clarify our sample sizes, as we believe the current manuscript may not have sufficiently highlighted the scale of our dataset. This study is based on 3,754 total observations, which is a dataset much larger than those we acquired in our past works. For context, our human-to-mouse translation study published in the Journal of Neuroscience relied on only 1,125

© 2026. Published by The Company of Biologists under the terms of the Creative Commons Attribution License (https://creativecommons.org/licenses/by/4.0/).

samples (Cottam et al., 2025). We consider this expanded dataset to be a major achievement to translate ages.

Specific modalities like MRI and blood work comprise only a small fraction of the total data we used to generate cross-species age alignments. For instance, the 18 MRI observations account for just 1.9% of the total feline-human time points. To ensure full transparency regarding these proportions, we updated Figure 1 to explicitly show the data source breakdown as requested by Reviewer 1.

In comparative aging research, sample attrition at advanced ages is a constraint. We are limited by the natural lifespan of cats (Teng et al., 2024) and by cats visiting the clinic. We have now included supplementary figure S1A, which shows the age distribution of pet cats visiting the Auburn clinic. You can see a sharp decline in the number of cats visiting the clinic past 12 years of age. Our datasets of MRI and bloodwork reflect the ages of cats available for study. We think including this information is important. Without it, readers may not appreciate the challenges of studying geriatric cats:

Age distribution of pet cats from the Auburn clinic

The ages of cats in this study were constrained by availability. There is a sharp decline in the number of cats visiting the clinic after 12 years of age (Fig. S1A). The inclusion of pet cats visiting the clinic permits studying individuals in their teens, but is also limited by the fact that relatively few individuals visit the clinic at advanced ages (Fig. S1A; Fig. 7, 8). We did not attempt to recruit aged cats for MRI studies because cats need to be sedated to acquire MRI scans, which carries a heightened risk of mortality in aged animals.

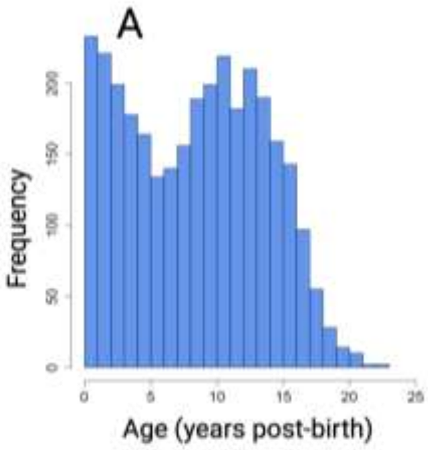

Figure S1. (A) Histogram of age of cats visiting the Auburn clinic. Cats visit the clinic in their teens, but there is a sharp fall in the number of cats visiting the clinic after 12 years of age, which makes sampling aged cats a challenge. For cats with multiple visits, we recorded the age at the last consultation…

References

Teng KT, Brodbelt DC, Church DB, O'Neill DG. Life tables of annual life expectancy and risk factors for mortality in cats in the UK. J Feline Med Surg. 2024 May;26(5):1098612X241234556. doi: 10.1177/1098612X241234556. PMID: 38714312; PMCID: PMC11156239.

Completeness

The narrow conclusion that cats and humans show broadly similar age-related changes in several gross brain metrics is supported reasonably well by the data presented. The paper also supports the claim that pet cats seen clinically tend to be older than colony cats, and therefore provide access to older feline ages than colonies typically do. But, the broad conclusion that this work "establishes cats as powerful and naturally occurring models for the study of neurodegeneration" is not

© 2026. Published by The Company of Biologists under the terms of the Creative Commons Attribution License (https://creativecommons.org/licenses/by/4.0/).

supported by the data shown. The study excludes overt lesions and tries to exclude cognitive dysfunction, but the cats are not phenotyped for longitudinal cognitive decline, neuropathological burden, or mechanistic similarity to human neurodegenerative disease. In fact, the authors explicitly concede that they cannot rule out cognitive dysfunction in some cats. That in itself seems to weaken the claim that the MRI changes clearly represent normal ageing rather than mixed ageing-plus-disease, and it certainly prevents a strong inference about modelling neurodegeneration.

We agree with the reviewer's suggestion, and we have revised the text to focus on the immediate implications of our results. We have reduced our attention to the broader 'One Health' context. Regarding the study of dementia, there are currently no well characterized methods to study age-related changes in memory performance in cats. While surveys exist to characterize cognitive dysfunction in pets (e.g., Landsberg et al., 2010; MacQuiddy et al., 2022), they often include behaviors, such as house-soiling. These behaviors are not directly related to memory performance. Such changes in behavior may arise due to health conditions other than cognitive dysfunction.

We agree that refining these cognitive tools is a vital next step, but developing and validating a new behavioral assessment framework is beyond the current scope of this study. Such an undertaking would require significant resources and separate large-scale funding. We believe this manuscript provides the necessary foundation for such investigations, and we have added a statement to the Discussion recommending this as a future direction.

We now state in the Discussion section:

"Because colony cats were not studied past their teens, we were not able to compare age-related brain atrophy between cat groups. We were not able to assess whether MRI, environment, or health status impacts rates of brain atrophy. Future work will be needed to identify whether environment, cognitive dysfunction, or genetic background may influence rates of brain atrophy across feline populations. We have much more to learn about feline cognitive dysfunction. Surveys and assessment tools exist to evaluate cognitive dysfunction in cats, but we have yet to develop cognitive assays that specifically test for memory impairment (Landsberg et al., 2010; Gunn-Moore, 2011; MacQuiddy et al., 2022). The development of such tests are needed before we can evaluate whether brain aging is accelerated in cats with cognitive dysfunction."

References

Landsberg GM, Denenberg S, Araujo JA. Cognitive dysfunction in cats: a syndrome we used to dismiss as 'old age'. Journal of feline medicine and surgery. 2010 Nov;12(11):837-48.

Gunn-Moore, D.A., 2011. Cognitive dysfunction in cats: clinical assessment and management. *Topics in companion animal medicine*, *26*(1), pp.17-24.

MacQuiddy B, Moreno J, Frank J, McGrath S. Survey of risk factors and frequency of clinical signs observed with feline cognitive dysfunction syndrome. Journal of feline medicine and surgery. 2022 Jun;24(6):e131-7.

There are also design issues that limit interpretation. First, the pet-versus-colony comparison is confounded by age distribution itself. The manuscript's conclusion is that pet cats show more atrophy largely because they are older, not because pet status per se changes brain ageing. That means the paper cannot distinguish environment, veterinary ascertainment, scanner differences, and ageing exposure cleanly.

To better compare biological aging in humans and cats, we include cats from colony and pet environments. Rather than viewing these populations as confounding variables, we consider the integration of colony and pet cats a primary strength of our study. This approach allowed us to synthesize data from diverse feline environments to achieve cross-species age translation that are relevant to both pet and colony cats. Notably, our colony samples did not exceed 10 years of age. The inclusion of pet cats seen in clinical settings was essential to capture age alignments towards late stages of life. Excluding these older pet cats to avoid confounds would have effectively precluded our ability to establish age equivalencies at late stages of life.Our previous comparative

© 2026. Published by The Company of Biologists under the terms of the Creative Commons Attribution License (https://creativecommons.org/licenses/by/4.0/).

work (e.g., chimpanzees and mice) indicates that most animals in laboratory settings rarely survive past the human equivalent of the eighth decade.

We have shifted our focus away from One Health, and we have expanded our focus on these issues raised by the reviewer. While we acknowledge that environmental, veterinary, and scanner-related variables cannot be fully disentangled, the age-related atrophies in cats remain compelling.

Second, the translational model is partly built on imputed missing observations across species. Without clear sensitivity analyses, how do we know how much the headline alignments depend on the imputation procedure rather than the observed data? Third, the study uses cross-sectional rather than longitudinal imaging. That is ok for initial trajectory fitting, but it means statements about within-animal ageing or rates of decline should be kept cautious. Fourth, the manuscript uses age-related blood chemistry and developmental milestones to support cross-species age alignment. That may be acceptable for a general framework, but those measures do not directly validate the cat as a model of human brain ageing or neurodegeneration.

The event scale used to generate the model to translate ages across species relies on imputed data. We include this model because it permits translating ages simultaneously across all species in the dataset. We used age from unimputed observations and those predicted from the model interchangeably. We have included (Figure S2A) to compare unimputed observations with those predicted values from the model. We include a description of this analysis in the Materials and Methods section.

The aim of our study is to translate ages across the lifespan of humans and cats, which necessitates amassing observations across the lifespan, including observations from young and old cats. We have refocused the manuscript to explain our focus on age translations across the lifespan. We reason these changes clarify why we collect information across the lifespan.

Finally, there are certainly missing experiments that could disprove the conclusion, in my view. The most important would be a longitudinal cognitive phenotyping linked to MRI in ageing cats with neuropathological confirmation in aged cats spanning cognitively normal and impaired animals, formal harmonisation or replication of MRI-derived morphometry in a scanner-controlled subset, and a clear demonstration that the cross-species mapping is robust to alternative model specifications and to removal of imputed data. None of these would be trivial, and the authors do acknowledge one limitation, namely that some cats may still have cognitive dysfunction despite screening. But they do not, in my view, adequately discuss the broader limitations of heterogeneity, imputation, and disease-versus-normal-ageing inference.

We agree that comparing cognitively impaired and healthy aging populations is a compelling future direction. Our primary conclusion is that cats reach biological milestones equivalent to human octogenarians. We do not see how the inclusion of cats with cognitive dysfunction would impact our ability to translate ages in cats and humans. Our understanding of feline cognitive dysfunction is still in its infancy. Behavioral surveys exist to assay cognitive dysfunction in cats, but they include general markers like house-soiling rather than specific memory impairments. We view our study as a vital foundation for this future work, which will require the development of specialized cognitive tests for cats.

We have included repeated measures from our colony cats (see Table S3). In our desire to anonymize cats, we omitted IDs from Table S3, containing brain volumetric results. We have assigned the cats numbers so you can see what MRI measures come from the same individual. We have reworded the text to clarify that scans may be acquired from the same individual.

Performing repeated MRI scans on geriatric pet cats presents significant ethical and practical challenges. Sedation carries a heightened risk of mortality in aged cats, and we believe pet owners would be, rightfully, unwilling to risk their animals' lives for the sake of the study. We have updated the Discussion to explicitly address these challenges. We also now add a section in the supplementary materials to show the age distribution of pet cats visiting the clinic.

Scholarship

© 2026. Published by The Company of Biologists under the terms of the Creative Commons Attribution License (https://creativecommons.org/licenses/by/4.0/).

The only sense in which the scholarship is weak is in discussing evidence that argues against the stronger translational conclusion. The paper does not seriously engage with the distinction between resemblance in gross structural ageing versus validated disease homology. It also does not adequately discuss literature on the limits of opportunistic clinical imaging cohorts, ascertainment bias in veterinary populations, or the confounding impact of mixed scanner/protocol data on morphometry. I also think that the advocacy, especially around One Health and the inadequacy of laboratory models, is creeping into over-reach given the presented evidence.

Thank you. We have reduced our focus on One Health.

Although we previously have in the methods section described the populations as belonging to the clinic. We now discuss multiple tests to make the reader aware that there are issues that may result from integrating information across multiple MRI scanners. We also state that we cannot evaluate how environment or MRI type impacts rates of aging.

We now state:

"Because colony cats were not studied past their teens, we were not able to compare age-related brain atrophy between cat groups. We were not able to assess whether MRI, environment, or health status impacts rates of brain atrophy. Future work will be needed to identify whether environment, cognitive dysfunction, or genetic background may influence rates of brain atrophy across feline populations. We have much more to learn about feline cognitive dysfunction. Surveys and assessment tools exist to evaluate cognitive dysfunction in cats, but we have yet to develop cognitive assays that specifically test for memory impairment (Landsberg et al., 2010; Gunn-Moore, 2011; MacQuiddy et al., 2022). The development of such tests are needed before we can evaluate whether brain aging is accelerated in cats with cognitive dysfunction."

Reviewer 2: The authors use a series of brain metrics from cats and humans, other health-related metrics from cats and humans, and other aging data from chimpanzees and mice, which were obtained from various sources (apparently from this and previous studies) to establish aligned developmental and aging trajectories across human and cat lifespans and propose that cats, most clearly pet cats in clinics could be a powerful model for the study of human aging, most notably brain aging. However, the way this potentially interesting article is written is not sufficiently clear and is non-linear, making it very difficult to follow and critically interpret. Figure 1, for instance, is presented at the very beginning, but the results of, say, Panel A (Figure 1A) are mostly described/dissected in the last sections of the Results. This and other methodology and data presentation issues described below makes reading the manuscript very confusing and time-consuming. Throughout the manuscript, it is hard to understand what corresponds to new data collected for the first time in this study, data extracted from previous publications, data computationally digitized from graphs in previous studies, and data that were imputed. How the different metric data were integrated is also not entirely clear. These points must be clarified to allow a full critical evaluation of experimental quality, sample quality and size, reproducibility, and the relevance and novelty of the analyses. In the current format, it is not possible to evaluate how well the title and abstract reflect the data and how well the authors discuss the merits of their data, without making assumptions or deductions. For instance, what data were used to obtain the graphs in Figure 1C? Figure 1C is not directly referred to in the text, nor are Figures 1B or 1D, for instance. I did not check the other figures.

Thank you for your critical feedback. We appreciate the time taken to read and provide feedback on our manuscript.

Table S1 provided a comprehensive list of all data sources used for our age-translation model as well as their sources. We recognize that the sheer volume of observations (N=3,754) may have obscured the overall data composition. To address the reviewer's concerns regarding clarity, we have substantially revised Figure 1 and split what was Figure into multiple figures. Figure 1 now includes a visual breakdown of these sources, ensuring the provenance of our dataset is accessible to the reader. We also include a relative breakdown of observations from MRIs and blood work in response to reviewer 1's concerns. As you can see, the MRI data accounts for 1.8% of observations (Figure 1).

© 2026. Published by The Company of Biologists under the terms of the Creative Commons Attribution License (https://creativecommons.org/licenses/by/4.0/).

We have added a section in the supplementary materials to provide additional explanations about the dataset. We also included additional validation analyses to showcase how model predictions compare with unimputed observations.

Abstract (and throughout the text)

- Some terms are poorly defined or used in loose ways. For instance, colony and pet cats. Abstract, line 31: "pet and colony cats", then in line 35 it mentions cats from clinics, zoos, and colonies. Please use precise nomenclature and/or explain what categories are equivalent.

We agree. Thank you for your close attention to our work. We have clarified these terms. We have replaced the terms “clinics, zoos, and colonies” with “pets, zoos, and colonies” in the abstract. We have modified terminology to be more consistent throughout the manuscript.

- Please clarify precisely what "colony cats" means; it is not clear to the average non-specialist reader. In the methods, please define "colony". Define what is the Auburn colony and Japan colony, and when "colony" is used alone in the text, please state what does it refer to.

Yes, thank you. We now include a definition of colony cats.

In the methods section (page 19), we now state “We studied colony cats, which are animals maintained in an enclosed area for research and breeding.”

In the abstract we have specified that colony cats can be used interchangeably with laboratory “colony (i.e., laboratory). We also specified that colony cats are laboratory cats and are also laboratory cats in the results section page 11 “colony (i.e., laboratory).”

We also reworded our reference to Auburn and Japan colonies to alleviate confusions. We refer to these colonies based on the institutes.

We now explain in better details the origin of the colony cats in the figure legend of figure 5. We now state:

“We first fit a smooth spline through these data followed by a non-linear model (see inset) to extract the age of plateau as well as epochs (e.g., 90 percent of plateau) within multiple cat groups, including colony cats from multiple sources, including one colony cats housed at the Toxicology laboratory in Kyoto, Japan (Nakai et al., 1992) and one housed in at Auburn University in the USA.”

- Specify whether the pet cats come from clinics only and/or zoos or elsewhere.

Yes, thank you. We should have defined these groups more clearly.

In the methods section, we now state that:

“Colony cats are defined as animals that are maintained in an enclosed area for research and breeding. Pet cats are companion animals that are studied in clinical, laboratory, shelter, or home settings. Zoo cats are managed for species preservation rather than for human companionship and are housed in enclosures.”

- In Figure 5, a new class appears: "clinics vs research". Please clarify what "research" means and ensure consistency throughout.

Yes, thank you. We understand this comment relates to figure 5 legend. We have renamed the clinic with pets.

Figures and figure legends

If possible, please provide more detail in the figures or figure legends about the data (n; whether values are imputed or observed); plots (box plots (and whiskers) and line plots—e.g., what the lines

© 2026. Published by The Company of Biologists under the terms of the Creative Commons Attribution License (https://creativecommons.org/licenses/by/4.0/).

and shaded regions represent (95% confidence intervals?)); and basic statistics for the fits (as approximately done for Panel D in Figure 6; although the box plot box and whiskers do not seem to be defined in that panel or in the methods).

The data are in Table S1.

Figure 5 legend, panel D: In the previously submitted version of the manuscript and in the current version of this manuscript, we explained that we applied a non-parametrics Welch one way test followed by a Games-Howell test.

We now include the following information in the figure 5 legend:

“Data are presented as box plots where the center line denotes the median, the box represents the interquartile range (25th to 75th percentiles), and the whiskers represent the minimum and maximum values.”.

- Page 7, line 135: "structures increase with age (e.g., interthalamic adhesion)…" Please verify. As interthalamic adhesion ultimately decreases with age, perhaps the authors meant something like "subarachnoid volume", which increases with age in both cats and humans?

Yes, thank you. We have fixed this typo.

- Lines 136-137: A word may be missing in "… time points in human and ??? (Figure 4)." Perhaps "cats"?

Yes, thank you. We have added the word “cats.”

- There are double punctuation marks after e.g., or i.e., in lines 132, 137, and 138. Please check these and verify elsewhere in the manuscript.

Thank you. We have addressed this issue.

Results

- Results, page 7 (quoted text):

"We used these smooth splines to extract corresponding time points in humans and (Figure 4). For example, we extracted the age at which the brain volume is maximal (i.e.., 100%) and when it reaches a percentage of its maximum size (e.g.., 90%, 80%) in the two species. We did this for multiple metrics to extract corresponding time points between humans and cats."
It is unclear how the corresponding time points from multiple metrics were integrated across metrics. Please clarify.

Thank you. We now appreciate that this statement was not clear, and we have now reworded it.

“We extracted the age at which the brain volume is maximal (i.e.., 100%) and when it reaches a percentage of its maximum size (e.g.., 90%, 80%) in the two species. We repeated this approach for the interthalamic adhesion area and thickness, which showed similar age-related changes in humans and cats.”

- Results, Figure 5: Please define ITA in the legend. Please provide statistical information (what the * indicates), describe the box plot (box and whiskers), and provide N. The relevant information appears to be in the text (consider moving it to the legend).

Yes, thanks. We now define ITA in the legend and state that * means that differences are statistically significant.

- Results, page 11, lines 185-190: The phrase "multiple sources" is not sufficiently precise to allow replication. Please specify the sources used (and verify this throughout the manuscript).

© 2026. Published by The Company of Biologists under the terms of the Creative Commons Attribution License (https://creativecommons.org/licenses/by/4.0/).

The sources are listed in Table S1 to allow replication. In the text, we now make reference to table S1. We consider it is not practical to list all the sources of 3,754s of observations. However, this information is listed in Table S1.

- Results, Figure 6:
- Panel A: Consider capitalising "A" in "Catage". I also suggest using a consistent font size across all figures to improve readability, if possible.
- Panel C: Please check "phosphotase", and note that the inset is barely readable.
- Panel E: Please check the y-axis label. Maybe include it, even if redundant with panel D.

Thank you for your feedback and careful attention to our figures. We have made the suggested changes.

Materials and Methods

- Lines 353-354: Please specify (at least in Table S1) exactly which data were extracted using WebPlotDigitizer v4 (Rohatgi, 2025), and the conditions used (e.g., manual vs automated extraction).

We did not take note of when we used the webplot digitizer to extract data. We do provide additional details. We now state:

"We used Web Plot digitizer v. 4 (Rohatgi, 2025) to extract some data from published plots, particularly for data points located between axis tick marks."

- Line 355: Typo: "we discussed howe..."

Thanks. We have fixed this typo.

- Line 501:
"The dataset contains missing data (Figure 1E; Table S1). We used the library package Amelia to generate imputed datasets (n=10)."
It is unclear what data are missing, which missing values were imputed, and in which datasets. The authors refer to Table S1, but it is unclear what is missing in Table S1. What is the relationship between missing data and "unknown" in Table S1? What was done when a statistic was "unknown"? What the authors did with these data is very hard to follow. Please provide more information. Please also provide information on what the library package Amelia is. If it is an R package, please state so and, if possible, provide reference(s) for the packages.

We have added Table S2, which reconfigures the data to display time points as rows and species-specific data as columns. Missing data points are explicitly marked as 'NA.' While Table S2 provides averaged information for each time point and for each species, we have retained Table S1 to ensure transparency regarding individual observations. Multiple observations (listed in Table S1) are averaged to generate a single time point (Table S2). Table S1 lists individual observations as well as their sources. We continue to provide our scripts to ensure that the reader can read through our imputation and alignment pipelines in R so that our analyses remain reproducible and accessible.

In the environment column, we list whether the individuals are from captive clients, captive laboratories, or unknown. In some cases, the environment is unknown or the environment was not always clearly articulated. We now explain that the environment from some individuals is not known in the Materials and Methods section. We explain what is in Table S1 and Table S2.

If a statistic was not known, we assumed it would be a 2 (i.e., midrange, mean or median). We should have specified this, and we have included this information in the Methods sections.

We now specify:

We used the library package Amelia, which is an R library package designed for imputation of incomplete multivariate datasets, to generate imputed datasets (n=10; Zhang, 2016).

© 2026. Published by The Company of Biologists under the terms of the Creative Commons Attribution License (https://creativecommons.org/licenses/by/4.0/).

Zhang Z (2016) Multiple imputation for time series data with Amelia package. *Annals of translational medicine*, *4*: 56.

Supplementary materials

- Table S1: Please include "This study" if/where the data is reported for the first time in this study, or describe from where it was extracted with a reference.

We have included references in Table S1. We now include “this study” for the MRI data, but we find it confusing to add “this study” to a separate reference if we acquired the data from another source. The datasets we used in the past are readily available either in past work or as dryad datasets. We have made our past and present datasets readily accessible, which enables the reader to identify what was collected in the past and what is part of the present study. We also labeled the type of data we acquired in Table S2, which may help alleviate confusion.

- Table S1 Legend: Missing a ")" at the end of the sentence. Please describe the "sex" categories—specifically, what does "male+female" mean?

Yes, we should have explained this category, thank you. We have updated the terminology to clarify that the 'Male+Female' designation is used for pooled data containing both sexes, as well as instances where the sex of the subjects was not specifically recorded. We now include this information in the Materials and Methods section.

- Table S3 Legend: Please provide units for all variables measured, where applicable.

Thanks. We should have provided units. We have now included units in the column name of Table S4 and the legend.

- Table S4 Legend: Missing "y" in "Summar".

Thank you for taking a careful look at our manuscript. We have fixed this typo.

- Please include a discussion of the effects of imputed data on the interpretations made. Please specify exactly which data were imputed in each section that uses or mentions imputed data.

Thank you. We now specify in the text that the observations in figure 7 are from unimputed observations. In the methods section, we reiterate that observations in figure 7 are from unimputed observations and those from the model in figure 8 rely on the event scale, which relies on imputed data. We also include an analysis that compares observations from imputed data with predicted values from the model (Fig. S2).

As part of an effort to improve clarity, we have now included Table S2, which has data rearranged from Table S1. Table S2 now spells out with an NA what data are missing.

Thank you for your feedback and careful attention to our work.

Second decision letter

MS ID#: bio.062604R1

MS Title: Cat brains age like humans: Translating Time shows pet cats live to be natural models for human aging

Authors: Christine Charvet, Capucine Januel, Elijah Morrow, Ryan Gibson, Amanda Gross, Alexandra A. de Sousa and Brier A. Rigby Dames

© 2026. Published by The Company of Biologists under the terms of the Creative Commons Attribution License (https://creativecommons.org/licenses/by/4.0/).

I am happy to tell you that your manuscript has been accepted for publication in Biology Open, pending our standard publication integrity checks. It was accepted on 7th May 2026.

© 2026. Published by The Company of Biologists under the terms of the Creative Commons Attribution License (https://creativecommons.org/licenses/by/4.0/).
